# Spontaneous and specific chemical cross-linking in live cells to capture and identify protein interactions

**DOI:** 10.1038/s41467-017-02409-z

**Published:** 2017-12-21

**Authors:** Bing Yang, Shibing Tang, Cheng Ma, Shang-Tong Li, Guang-Can Shao, Bobo Dang, William F. DeGrado, Meng-Qiu Dong, Peng George Wang, Sheng Ding, Lei Wang

**Affiliations:** 1 Department of Pharmaceutical Chemistry and the Cardiovascular Research Institute, University of California San Francisco, 555 Mission Bay Blvd. South, San Francisco, California 94158 USA; 2 Gladstone Institute of Cardiovascular Disease and Department of Pharmaceutical Chemistry, University of California San Francisco, 1650 Owens St., San Francisco, California 94158 USA; 3 Department of Chemistry and Center for Therapeutics and Diagnostics, Georgia State University, P.O. Box 3965, Atlanta, Georgia 30302 USA; 4 National Institute of Biological Sciences, 7 Science Park Rd., Beijing, 102206 China

## Abstract

Covalently locking interacting proteins in situ is an attractive strategy for addressing the challenge of identifying weak and transient protein interactions, yet it is demanding to execute chemical reactions in live systems in a biocompatible, specific, and autonomous manner. Harnessing proximity-enabled reactivity of an unnatural amino acid incorporated in the bait toward a target residue of unknown proteins, here we genetically encode chemical cross-linkers (GECX) to cross-link interacting proteins spontaneously and selectively in live cells. Obviating an external trigger for reactivity and affording residue specificity, GECX enables the capture of low-affinity protein binding (affibody with Z protein), elusive enzyme-substrate interaction (ubiquitin-conjugating enzyme UBE2D3 with substrate PCNA), and endogenous proteins interacting with thioredoxin in *E. coli* cells, allowing for mass spectrometric identification of interacting proteins and crosslinking sites. This live cell chemistry-based approach should be valuable for investigating currently intangible protein interactions in vivo for better understanding of biology in physiological settings.

## Abstract (web summary)

Proteins associate via weak and transient interactions that are challenging to identify in vivo. Here, the authors use a genetically encoded chemical cross-linker to covalently lock interacting proteins in live cells, allowing them to identify the captured proteins by mass spectrometry.

## Introduction

Proteins interact with each other to perform various biological functions, and identification of protein interactions in their physiological setting is critical to understanding molecular mechanisms of biological processes and pathophysiology. In addition to approaches using non-covalent binding such as affinity purification and immunoprecipitation^1^, methods based on covalent bonding have been gaining strong momentum in recent years, as the stable covalent linkage irreversibly locks the interacting proteins and tolerates stringent processing, thus potentially enabling the identification of weak and transient interactions with higher specificity, reliability, and accuracy^2–4^. One method involves genetically encoding photo-crosslinkers into proteins, which upon UV light-activation react with nearby residues to crosslink interacting partners^5–12^. Reactivities of the activated photo-crosslinkers are non-selective toward amino acid residues, allowing any protein nearby to be cross-linked. However, such non-selectivity increases background crosslinking, makes crosslinking sites unpredictable, and thus dramatically complicates mass spectrometric analysis^13^. Photo-activated crosslinkers can also be readily quenched or rearrange to become inactive, resulting in short half-life^3, 4^; capture of weak and transient interactions within the short reactive window sufficient for identification thus remains challenging. Another method uses small molecules to chemically crosslink certain type of residues (e.g., lysine) in pairs on protein surfaces^14–19^. Coupled with mass spectrometric analysis^20^ of the cross-linked complex, information is gained for protein structure and interaction based on residue-specific reactivity. However, highly reactive small molecules are needed for cross-linking, making it challenging to apply to live systems. Simultaneous reacting with all targeted residues in all proteins also generates excess number of covalent inter- and intra-protein bonds, which may artificially alter protein conformation or interaction^14^. Application of small molecule chemical cross-linkers has been mainly limited to large protein complexes with stable interactions. Therefore, despite progress aforementioned, methods capable of capturing and identifying weak and transient protein interactions in live cells remain challenging and desirable.

To covalently capture interacting proteins in live cells, biocompatible chemistry is needed to target unmodified natural proteins under mild physiological conditions and with high specificity. The reaction preferably occurs without an exogenous trigger to obviate timing and to facilitate in vivo application. Fulfilling these stringent demands, here we report a method, Genetically Encoded Chemical Cross-linking of proteins coupled with Mass Spectrometry (GECX-MS), to identify protein–protein interactions in live cells. An unnatural amino acid (Uaa) with proximity-enabled bioreactivity is genetically incorporated into the bait protein, which reacts with a specific residue of the interacting protein upon protein–protein interaction, thus covalently cross-linking the interacting protein with the bait protein for MS identification. This method integrates the advantages of genetic encoding to achieve live cell compatibility and of chemical cross-linking to achieve spontaneous reactivity and residue specificity. Harnessing these unique abilities, we demonstrate the capture and identification of low-affinity protein binding, elusive enzyme-substrate interactions, and endogenous proteins interacting with thioredoxin directly in live *E. coli* cells.

## Results

### GECX based on proximity-enabled bioreactivity

The reactivity of photo-crosslinkers is latent until light activation, allowing them to be genetically encoded for use in live cells through the expansion of the genetic code^5, 21^. In contrast, chemical cross-linkers possess constant reactivity, and thus have been infeasible to genetically encode, as such reactivity, if uncontrolled, will result in non-specific crosslinks with the ubiquitously present target amino acid and potentially cause cellular toxicity. We recently developed proximity-enabled bioreactivity, in which a genetically encoded Uaa selectively reacts with a target natural amino acid residue only when the two residues are in proximity^10, 22–25^. Here we expand this concept in vivo to genetically encode spontaneous chemical cross-linkers for identifying protein–protein interactions in live cells.

**Fig. 1:**
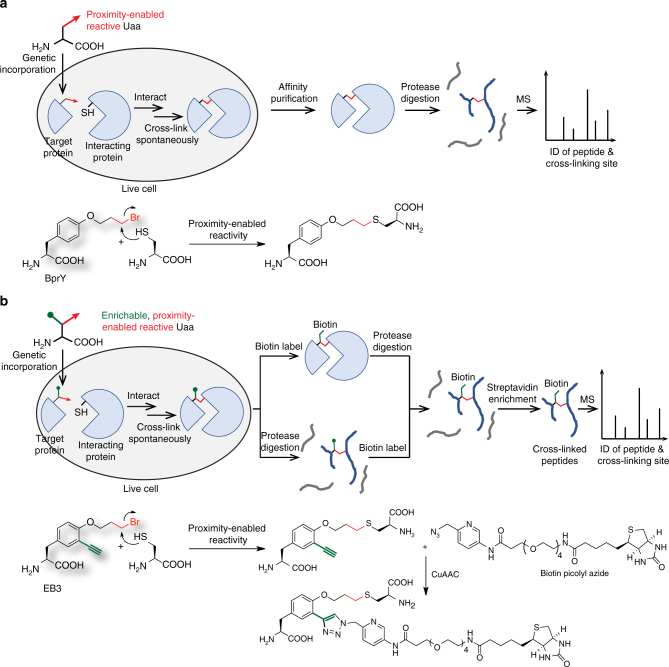
GECX to capture protein interactions in live cells for MS identification. **a** Schematic illustration of the GECX-MS strategy. The Genetically Encoded Chemical Cross-linking (GECX) Uaa has proximity-enabled reactivity, which specifically reacts with the target residue (e.g., Cys) of interacting proteins only when the two proteins interact and place the Uaa in proximity to the target residue. The resultant covalent linkage captures interacting proteins in situ for subsequent identification with Mass Spectrometry (MS). The structure of the chemical cross-linking Uaa BprY and its reaction with Cys are shown at the bottom. **b** Schematic illustration of GECX-MS using the enrichable chemical cross-linking Uaa EB3. Structure of EB3 and its reaction with Cys via proximity-enabled reactivity, followed by labeling with biotin through the click chemistry CuAAC are shown at the bottom

Specifically, we designed a chemical crosslinking Uaa BprY (Fig. 1a), which contains a proximity-enabled reactive group, alkyl bromide, for chemical cross-linking. The alkyl bromide of BprY would react with a Cys of interacting proteins only when the Uaa is in close proximity to the target Cys, generating a covalent linkage between interacting proteins for capture and identification. Cells expressing interacting proteins are cultured with BprY. The Uaa is genetically incorporated into the target protein at the desired site, and it reacts with Cys of interacting proteins to directly capture the protein–protein interaction in live cells. After affinity purification of the target protein, the co-purified cross-linked protein is digested by protease and analyzed with mass spectrometry to reveal the protein identity and crosslinking site.

An enrichable chemical cross-linking Uaa, EB3, was also designed (Fig. 1b). In addition to the alkyl bromide group for chemical crosslinking, EB3 contains an extra bioorthogonally reactive group for enrichment: the alkyne group is harnessed to label biotin for enriching cross-linked peptides to improve detection sensitivity and accuracy. Similar to BprY, EB3 is also genetically incorporated into the target protein to capture protein–protein interaction in live cells (Fig. 1b). After affinity purification of the target protein, the co-purified cross-linked protein sample is then biotinylated via the biorthogonal alkyne group, either before or after protease digestion. Digested cross-linked peptides are enriched with streptavidin beads and analyzed with mass spectrometry to identify the interacting protein and the cross-linking site.

### Genetic encoding chemical crosslinking Uaa and EB3 labeling

We previously reported the synthesis of Uaa BprY and the evolution of an orthogonal tRNA^Pyl^_CUA_/MmXYRS pair for genetic incorporation of BprY into proteins via suppression of the amber stop codon^26^. The enrichable Uaa EB3 was synthesized in 5 steps as described in detail in Supplementary methods and Supplementary Fig. 1. EB3 structurally differs from BprY (Fig. 1) by an alkyne group only, so we hypothesized that the orthogonal tRNA^Pyl^_CUA_/MmXYRS should be able to incorporate EB3 as well. To test this possibility, we co-expressed the tRNA^Pyl^_CUA_/MmXYRS pair with the gene encoding an affibody^27^ containing a TAG codon at site 36 and a C-terminal His6 tag (Afb_36TAG_His6) in *E. coli* DH10β cells. In the presence of EB3 in cell culture, full-length affibody protein was produced, and MALDI-TOF mass spectrometric analysis of the purified protein confirmed EB3 incorporation (Supplementary Fig. 2a, d). To further validate EB3 incorporation, we also expressed the gene encoding maltose binding protein (MBP) fused with Z protein containing a TAG codon at site 24 (MBP-Z_24TAG) together with tRNA^Pyl^_CUA_/MmXYRS in *E. coli* DH10β cells. As shown by SDS-PAGE and western analysis (Supplementary Fig. 2b, c), in the presence of EB3 full-length MBP-Z fusion protein was produced in the yield of 2 mg/L. High resolution tandem mass spectrometric analysis of the expressed protein (Supplementary Fig. 2e) indicated that only EB3 and no other amino acids was selectively incorporated at the TAG site. These results demonstrate that the tRNA^Pyl^_CUA_/MmXYRS pair was able to site specifically incorporate the Uaa EB3 into proteins in response to the TAG codon in *E. coli*.

We next tested whether the alkyne group of EB3 would be suitable for attaching biotin via copper-mediated azide–alkyne Huisgen cycloaddition (CuAAC). Uaa EB3 was incubated with an azide-functionalized biotin, biotin picolyl azide (Fig. 1b), and high-resolution mass spectra demonstrated biotinylation of EB3 on the free amino acid (Supplementary Fig. 3a, b). We then incubated the biotin picolyl azide with EB3-incorporated affibody protein. MALDI-TOF MS analysis of the reaction product (Supplementary Fig. 3d) in comparison to the EB3-incorporated affibody (Supplementary Fig. 3c) clearly showed that biotin was attached to the EB3-containing affibody protein.

### GECX to crosslink affibody with Z protein in *E. coli*

**Fig. 2:**
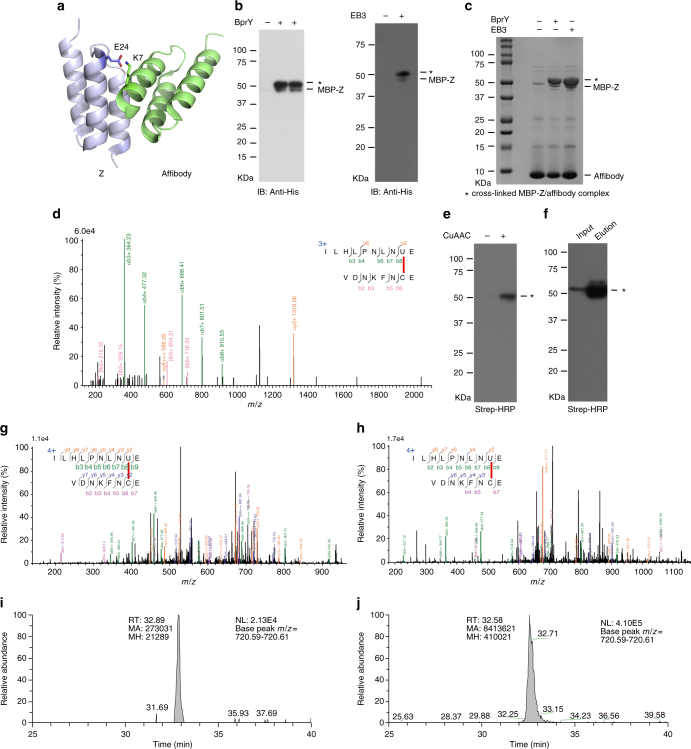
GECX-MS to capture and identify affibody binding to Z in *E. coli*. **a** Structure of the affibody–Z protein complex (PDB ID 1LP1), with two proximal sites, Lys7 in the affibody and Glu24 in the Z protein, highlighted. **b** Western blot of cell lysate of cells co-expressing MPB-Z(24BprY) with affibody(7Cys) and of cells co-expressing MPB-Z(24EB3) with affibody(7Cys). The cross-linked MBP–Z/affibody complex is indicated with a star. **c** SDS-PAGE of proteins His-tag purified from cells co-expressing affibody(7Cys) with MPB-Z(24BprY), or with MBP-Z(24EB3). In the absence of Uaa, background suppression of the TAG codon at site 24 resulted in small amount of full-length MBP–Z. **d** Mass spectrum of the cross-linked peptide between affibody(7Cys) and MBP-Z(24BprY). U represents BprY in the peptide sequence. **e** His-tag purified affibody(7Cys)/MBP-Z(24EB3) was labeled with biotin via CuAAC and then western blotted with streptavidin-HRP conjugate. **f** Biotin based enrichment of cross-linked affibody(7Cys)/MBP-Z(24EB3). **g** Mass spectrum of the cross-linked peptide between affibody(7Cys) and MBP-Z(24EB3) before biotin labeling. U represents EB3 in the peptide sequence. **h** Mass spectrum of biotinylated cross-linked peptide between affibody(7Cys) and MBP-Z(24EB3). U represents EB3 in the peptide sequence. **i** Extracted ion chromatography of biotinylated cross-linked peptide from input sample of biotin enrichment. **j** Extracted ion chromatography of biotinylated cross-linked peptide from elute sample of biotin enrichment. RT retention time, MA peak area, MH peak height

To determine the chemical cross-linking ability of BprY and EB3 via proximity-enabled reactivity in live cells, we co-expressed the prototypical protein affibody and its substrate Z protein^27^ in *E. coli* and tested whether the Uaa incorporated into the Z protein could cross-link a proximal Cys residue introduced into the affibody directly in *E. coli* cells. The affibody binds its substrate Z protein in relatively low affinity and with a *K*_d_ of 6 μM. On the basis of the crystal structure of the affibody–Z protein complex^27^, we introduced the chemical cross-linking Uaa at site 24 of the Z protein and Cys at site 7 of the affibody, placing the two residues in proximity upon protein interaction (Fig. 2a). MBP was fused to the Z protein to better distinguish the Z protein from the affibody as they have similar molecular weights (MW). Genes encoding affibody_7C_His6 and MBP-Z_24TAG_His6 were co-expressed with the tRNA^Pyl^_CUA_/MmXYRS pair in *E. coli* DH10β cells in the presence of BprY or EB3. After expression, cells were pelleted, boiled, and cell lysate analyzed with western blot (Fig. 2b). A strong cross-linking band with MW corresponding to MBP-Z/affibody complex was observed for both BprY and EB3. Lysing cells in native lysis buffer with 10 mM dithiothreitol followed by His-tag purification also showed a strong cross-linking band at the same position on SDS-PAGE gel for both Uaas (Fig. 2c). On the basis of band intensities for the cross-linked complex and MBP-Z, the cross-linking efficiency was 82% for BprY and 85% for EB3.

For comparison, we also incorporated the widely used photo-crosslinking Uaa *p*-azidophenylalanine (Azi) into MBP-Z at the same position as BprY and EB3, and performed photo-crosslinking of MPB-Z with affibody in *E. coli*. Only a faint cross-linking band was detected (Supplementary Fig. 4a-c) with a cross-linking efficiency of 10%. To understand the low-photo-crosslinking efficiency of Azi, we systematically analyzed the Azi-mediated photo-cross-linked affibody/MBP-Z sample with tandem mass spectrometry. Several products representing unsuccessful affibody/MBP-Z cross-linking were identified: Azi incorporated peptide corresponding to Azi unactivated by light (1%, Supplementary Fig. 5a, b); Azi-2N + 2 H peptide (77%, Supplementary Fig. 5c, d) and Azi-2N-2 H peptide (22%, Supplementary Fig. 5e, f) corresponding to photoactivated but non-cross-linked Azi. These side products could arise from the relatively low affinity of affibody/MBP-Z interaction and the short lifetime of nitrene^3, 4^, the reactive species of Azi upon photoactivation, and thus resulted in low-photo-crosslinking efficiency of Azi. Another photo-crosslinking Uaa *p*-benzoylphenylalanine (Bpa) was similarly tested at the same position as BprY and EB3, which cross-linked MBP-Z with affibody in *E. coli* with a crosslinking efficiency of less than 5% (Supplementary Fig. 4d–f). These results indicate that the in vivo chemical cross-linking efficiency of BprY or EB3 is much higher than the photo-crosslinking efficiency afforded by Azi or Bpa.

Using tandem mass spectrometry, we analyzed the protein sample His-tag purified from *E. coli* cells, wherein affibody was chemically cross-linked to MBP-Z via the genetically incorporated BprY. As shown in Fig. 2d, the BprY-containing peptide of MBP-Z was identified to clearly cross-link with the peptide of affibody, and the cross-linking site was precisely mapped to Cys7 in the affibody as expected.

For *E. coli* samples cross-linked using EB3, we attached biotin onto EB3 incorporated in MBP-Z via CuAAC, and enriched the cross-linked affibody/MBP-Z product using streptavidin for mass spectrometric analysis. The click CuAAC reaction to label EB3 with biotin was carried out on proteins His-tag purified from cells expressing affibody_7C_His6 and MBP-Z_24EB3_His6. Western blot analysis showed that biotin was successfully attached onto the cross-linked affibody/MBP-Z complex, and enabled the enrichment of the complex (Fig. 2e, f). Using tandem mass spectrometry, the biotinylated cross-linked peptide was identified from the cross-linked affibody/MBP-Z samples both before and after streptavidin enrichment. The cross-linking site between affibody_7C_His6 and MBP-Z_24EB3_His6 was also precisely mapped to Cys7 in the affibody (Fig. 2g, h). After enrichment, the intensity of the cross-linked peptide was 30 times more than the input sample (Fig. 2i, j).

To further confirm in vivo chemical cross-linking and biotin labeling via EB3, we also tested another pair of proximal sites in affibody and MBP-Z and switched EB3 into the affibody (Supplementary Fig. 6). EB3 was incorporated at site 36 of affibody and a Cys was introduced at site 6 of MBP-Z. Similarly, cross-linking of the affibody to MBP-Z in *E. coli* was detected, and biotin was labeled onto the cross-linked complex. Mass spectrometric analysis of the cross-linked product clearly mapped that EB3 of affibody cross-linked to Cys6 of MBP-Z. Taken together, these results indicate that GECX enables proteins binding in low affinity to be chemically cross-linked directly in live *E. coli* cells with efficiency higher than photocrosslinkers and to be identified by mass spectrometry with crosslinking site precisely mapped.

### GECX to crosslink enzyme UBE2D3 with substrate protein PCNA

A challenge in identification of protein–protein interactions in vivo is to detect weak and transient interactions such as those between enzymes and substrates. To assess whether GECX can address this challenge, we applied GECX-MS to characterize the interaction of the ubiquitin-conjugating enzyme UBE2D3 with its substrate protein PCNA (proliferating cell nuclear antigen) in *E. coli* cells. UbcH5C, a yeast E2 ubiquitin-conjugating enzyme, has been reported to ubiquitinate yeast PCNA (yPCNA) in vitro^28^. We hypothesized and confirmed that the human homolog, UBE2D3, could also ubiquitinate yPCNA in vitro using ubiquitination assay (Supplementary Fig. 7).

We first tested whether the interaction of UBE2D3 with yPCNA could be cross-linked in vitro with the small molecule chemical crosslinking approach. The widely used amine reactive small molecule crosslinker bis(sulfosuccinimidyl) suberate (BS^3^) was applied to the mixture of purified UBE2D3 and yPCNA using established protocols^29, 30^. However, no crosslinking of UBE2D3 to yPCNA was detected either by SDS-PAGE or western blot (Supplementary Fig. 8), which is consistent with our experience that enzyme–substrate interactions are generally elusive to this method.

**Fig. 3:**
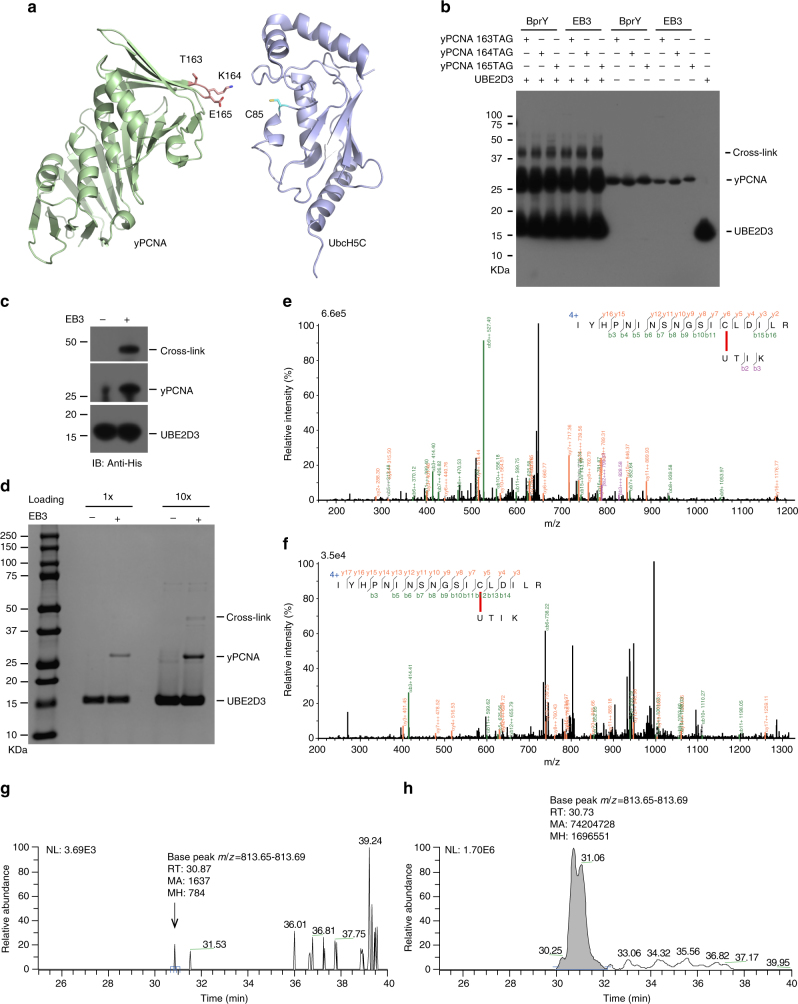
GECX-MS to capture and identify UBE2D3 interacting with PCNA. **a** Structure of the substrate protein yPCNA (PDB ID: 4YHR) with its ubiquitination site K164 and two neighboring residues T163 and E165 shown in stick; structure of UbcH5C (PDB ID: 1 × 23), a yeast homolog of UBE2D3 (a human ubiquitin E2 enzyme), is shown at right with the active site residue Cys85 highlighted in stick. **b** Western blot analysis of in vitro crosslinking of UBE2D3 to yPCNA via the genetically encoded Uaa BprY or EB3 incorporated into yPCNA. An anti-His6 antibody was used to detect the His6 tag appended at the C-termini of yPCNA and UBE2D3. **c** Western blot of cell lysate of cells co-expressing yPCNA(165EB3) and UBE2D3, showing the in vivo crosslinking of UBE2D3 onto yPCNA by EB3. **d** SDS-PAGE gel of His-tag purified proteins from cells co-expressing yPCNA(165EB3) and UBE2D3. **e** Mass spectrum of cross-linked peptide between yPCNA and UBE2D3. U represents EB3 in the peptide sequence. **f** Mass spectrum of biotinylated cross-linked peptide between yPCNA and UBE2D3. U represents EB3 in the peptide sequence. **g** Extracted ion chromatography of biotinylated cross-linked peptide from input sample of biotin enrichment. RT: retention time; MA peak area, MH peak height. **h** Extracted ion chromatography of biotinylated cross-linked peptide from elute sample of biotin enrichment

We then examined whether the genetically encoded BprY and EB3 could crosslink substrate yPCNA to enzyme UBE2D3 in vitro. BprY or EB3 was individually incorporated into yPCNA at residue K164, the ubiquitination site, and two neighboring sites T163 and E165 (Fig. 3a). The purified Uaa-incorporated yPCNA was incubated with the purified wild-type UBE2D3, and the mixture was analyzed using denatured SDS-PAGE. A crosslinking band corresponding to the covalent yPCNA/UBE2D3 complex was clearly detected (Fig. 3b) for all three sites tested; BprY and EB3 showed similar ability to crosslink yPCNA to UBE2D3 in vitro.

We next determined whether the genetically encoded chemical crosslinking Uaa could crosslink substrate yPCNA to enzyme UBE2D3 directly in *E. coli* cells. Since BprY and EB3 showed similar ability to crosslink affibody to MBP-Z in *E. coli* cells and to crosslink UBE2D3 to yPCNA in vitro, we proceeded with EB3 in *E. coli* cells. His-tagged UBE2D3 and yPCNA_165EB3 was co-expressed in *E. coli*. Cell lysate was analyzed by western blot, and His-tag purified proteins were analyzed by SDS-PAGE. Both western blot and SDS-PAGE gel showed that the two proteins were indeed cross-linked in live cells (Fig. 3c, d).

To confirm the crosslinking and to identify the crosslinking site, we analyzed the His-tag purified proteins with mass spectrometry. As expected, the cross-linked peptides were identified to be the yPCNA peptide containing EB3 at site 165 and the UBE2D3 peptide containing Cys85, the Cys in the activate site of UBE2D3 (Fig. 3e). No other peptide cross-linked to EB3 was detected, indicating that yPCNA was cross-linked to UBE2D3 specifically.

Although the cross-linked protein complex could be enriched by traditional affinity tags appended on the bait protein, the detection sensitivity of MS directly depends on the intensity of the cross-linked peptide fragment, not on the whole protein complex. The genetically encoded EB3, while being responsible for the crosslinking, also affords a unique avenue for direct enrichment of the cross-linked peptide via the alkyne functionality. To explore this ability of EB3, we attempted to directly enrich the cross-linked peptide through biotin labeling. We digested the His-tag-purified proteins using trypsin, labeled biotin onto EB3 via CuAAC, and enriched the cross-linked peptide using streptavidin pull-down. Analysis of this enriched sample showed the cross-linked peptide with biotin-labeled on EB3 and pinpointed the crosslinking site to Cys85 of UBE2DE (Fig. 3f), as expected. The intensity of the crosslinked peptide in the extracted ion chromatogram was strikingly higher than that before enrichment (Fig. 3g, h). Such dramatic increase in MS signal of the crosslinked peptide would be beneficial for detecting proteins with low expression. Collectively, these results indicate that the genetically encoded EB3 enabled the chemical crosslinking of an enzyme to its substrate in live *E. coli* cells, for which the captured enzyme, its crosslinked peptide, and the crosslinking site can be identified unambiguously.

### GECX-MS to identify Trx-interacting proteins in *E. coli*

**Fig. 4:**
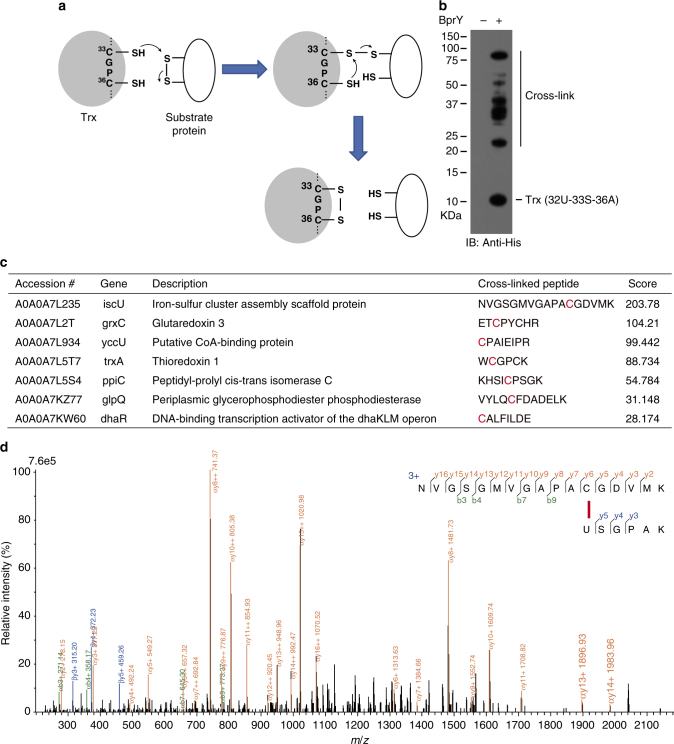
GECX-MS to capture and identify Trx-interacting proteins in *E. coli*. **a** Schematic illustration of disulfide reduction by thioredoxin (Trx). **b** Western blot of cell lysate of *E. coli* cells expressing Trx1(32BprY-33S-36A), showing multiple endogenous proteins crosslinked to Trx1. **c** List of proteins crosslinked to Trx1(32BprY-33S-36A) in *E. coli* cells that have the crosslinked peptides identified. The crosslinked Cys residues are highlighted in red. **d** Mass spectrum of cross-linked peptide between iscU and Trx1. U represents BprY in the peptide sequence. The mass spectra for other crosslinked proteins are provided in Supplementary Figure 9

We next explored whether GECX could be used to capture and identify unknown protein interactions in live cells. Thioredoxin (Trx) is a ubiquitous oxidoreductase that catalyze the reduction of disulfide bonds of proteins, serving as defense against oxidative stress and regulating various signal transduction pathways^31^. Trx proteins share a CGPC catalytic motif. The first Cys attacks an oxidized substrate, forming a mixed-disulfide intermediate between Trx and its substrate; the second Cys then attacks the first Cys to release the substrate in the reduced form and Trx in the oxidized state (Fig. 4a). Trx also involves in the reduction of sensitive Cys residues prone to oxidation by reactive oxygen species.

To identify proteins interacting with Trx in *E. coli* cells, we incorporated BprY into *E. coli* Trx1 at residue 32, a site adjacent to the first catalytic Cys, and mutated Cys33 into Ser and Cys36 into Ala to prevent possible intramolecular BprY crosslinking. Mutation of these Cys residues to Ser or Ala does not impair Trx folding and is often used in studying Trx function^31^. After expressing Trx1 (32U-33S-36A) in *E. coli*, we detected multiple new bands with MW higher than Trx1 on western blot using anti-His antibody, suggesting crosslinking of Trx1 to endogenous interacting proteins (Fig. 4b). Trx1 (32U-33S-36A) and its covalently crosslinked partners were purified by Ni-NTA affinity chromatography and subjected to mass spectrometric analysis. A total of 91 Cys-containing proteins that have two or more peptides in the mass spectra were identified (Supplementary Table 1). Among these identified proteins, MsrA, MsrC, Tpx, and AhpC are well-known substrate proteins of *E. coli* Trx1^31^, as previously demonstrated by other methods, which nicely validate the GECX-MS approach. Trx is ubiquitously present in various organisms with functional conservation. Our identified proteins also include 16 proteins that interact with Trx in other organisms (Supplementary Table 1). The remaining proteins could be new targets of Trx1 in *E. coli*.

GECX has a unique ability to form an irreversible covalent linkage between the interacting proteins in live cells. Since the crosslinking reaction occurs only upon Uaa in contact with the target amino acid^10, 22^, which requires two proteins interact, identification of the crosslinked peptide would provide direct evidence of protein interaction. Indeed, GECX-MS revealed 7 endogenous *E. coli* proteins (Fig. 4c), which all had a Cys residue crosslinked with BprY on Trx1, as clearly shown by tandem mass spectra (Fig. 4d and Supplementary Fig. 9). Among these proteins with crosslinked peptide identified, iscU and grxC are known to interact with Trx1^32, 33^. Crosslinking with endogenous Trx1 (encoded by trxA gene) suggests that Trx1 in *E. coli* might form dimers, and dimerization of Trx has been reported in the shrimp *Litopenaeus vannamei* ^34^. ppiC, yccU, glpQ, and dhaR have not been reported before to interact with Trx, the biological significance of which warrant further studies. In sum, these results show that GECX is able to capture unknown protein interactions in live cells for MS identification of the interacting proteins and mapping of the crosslinking sites.

## Discussion

By genetically encoding a Uaa reactive toward Cys in proximity, we developed GECX-MS to chemically crosslink interacting proteins with residue specificity directly in live cells for mass spectrometric identification. Both protein binding and enzyme–substrate interaction were successfully captured in vivo for MS detection, and the captured protein and crosslinking site were pinpointed unambiguously. This work demonstrates the initial feasibility of genetically encoding a chemical crosslinker for identifying protein–protein interactions in live cells.

In comparison with genetically encoded photo-crosslinkers, GECX spontaneously crosslinks interacting proteins, obviating an exogenous trigger (e.g. light) to activate the reactivity. Although temporal control is absent, protein interactions can be captured whenever they occur and no timing is needed to activate the crosslinking reaction, which should facilitate in vivo application where exogenous triggers are difficult to deliver or timing for trigger is hard to decide (which is common for transient interactions). In addition, upon activation photo-crosslinkers are often quenched or rearranged, shortening the time window to crosslink interacting proteins, whereas chemical crosslinkers used in GECX are constitutively reactive toward the target, accumulating crosslinked products until reacted and thus increasing the crosslinking efficiency. Moreover, while photo-crosslinking produces unpredictable crosslinking sites due to nonspecific reactivity, GECX generates predictable and defined covalent linkages facilitating mass spectrometric analysis.

Small molecule chemical crosslinkers are highly reactive and generally incompatible with live cells, while GECX has fine-tuned reactivity controlled by proximity and is suitable for use in live systems^10, 22, 25^. Small molecule chemical crosslinkers crosslink all proteins non-selectively and generates excess number of covalent bonds, but GECX genetically encodes the chemical crosslinker to afford protein and site specificity. Large number of covalent linkages could artificially alter proteins and complicates analysis^14^, while defined covalent bonds generated in GECX minimize potential interference, significantly simplify mass spectrometric analysis, and improve identification accuracy. Small molecule chemical crosslinkers have successfully identified stable proteins interactions, but remain challenging to capture weak and transient interactions and interactions among small proteins, wherein target residue pairs with appropriate distance are scarce. We show here that GECX is able to overcome both challenges.

GECX-MS is expected to have broad applicability. BprY and EB3 are reactive toward Cys in proximity, and Cys is present in proteins and enzymes involved in a myriad of signal processes, such as ubiquitination, phosphorylation, and nitrosylation, potentially allowing GECX-MS to dissect entangling substrate–enzyme specificities in these pathways and to identify potential drug targets in related pathophysiology. In addition, GECX-MS is not limited to targeting Cys. Uaas reactive toward other amino acids including Lys and His have also been genetically encoded^25, 35, 36^, which should expand the range of proteins addressable by GECX.

In conclusion, GECX-MS is compatible with live cells, able to capture and covalently lock interacting proteins in vivo, and directly enriches the crosslinked peptides for sensitive detection. The MS identification of the crosslinked peptide also serves as direct evidence for protein–protein interaction, whereas in methods based on non-covalent interactions, the identified proteins can be indirect interaction and false positive. We thus expect GECX-MS to be valuable for identifying weak and transient protein interactions in their native cell setting for a more physiologically relevant understanding. Lastly, this work highlights the feasibility of executing chemistry on proteins in live cells with biocompatibility and specificity, which should inspire further development and application of biocompatible chemistries to address the needs unfulfillable by bio-orthogonal chemistries.

## Methods

### Incorporation of EB3 into MBP-Z, affibody, yPCNA, and Trx1

For single protein purification (Supplementary Figs. 2, 7, and  8), genes for MBP-Z(Glu24TAG), affibody (Asp36TAG), and yPCNA(Thr163TAG, Lys164TAG, or Glu165TAG) were respectively cloned into the expression plasmid pBAD. For in vivo crosslinking, genes for MBP-Z (Glu24TAG) and WT affibody, for WT MBP-Z and affibody(Asp36TAG), and for yPCNA (Thr163TAG, Lys164TAG, or Glu165TAG) and WT UBE2D3 were, respectively, cloned into the pETDuet-1 vector. Trx1 (Trp32TAG, Cys33Ser and Cys36Ala) was cloned into expression plasmid pBAD. The resultant expression plasmids were individually co-transformed with plasmid pEvol-MmXYRS (expressing tRNA^Pyl^_CUA_/MmXYRS) into *E. coli* BL21 cells. A single colony was picked and cultured in 4 mL 2×YT media supplemented with 34 μg mL^−1^ chloramphenicol (Cm) and 100 μg mL^−1^ ampicillin (Amp) overnight. Aliquot of this startup culture was diluted into 100 mL of 2×YT media with antibiotics (OD_600_ < 0.1). When OD_600_ of cell culture reached 0.4, EB3 dissolved in DMSO was added at final concentration of 1 mM. Protein expression was induced by IPTG (for pETDuet-1 plasmids) at final concentration of 0.5 mM or by arabinose (for pBAD plasmids) at final concentration of 0.1% when OD_600_ of cell culture was around 0.6. After 6 h growth at 30 °C, cells were pelleted and frozen at −80 °C.

### His-tag protein purification

Cell pellet was resuspended in 14 mL lysis buffer (50 mM Tris-HCl pH8.0, 500 mM NaCl, 20 mM imidazole, 1%v/v Tween20, 10%v/v glycerol, lysozyme 1 mg mL^−1^, DNase 0.1 mg mL^−1^) with protease inhibitors. The suspension was incubated at 4 °C for 30 min with stirring. Lysate was sonicated with Sonic Dismembrator (Fisher Scientific, 30% output, 3 min, 1 s off, 1 s on) in an ice-water bath, followed by centrifugation (20,000, 30 min) to collect the supernatant. Protino^®^Ni-NTA Agarose slurry (80 μL) was rinsed 3 times with wash buffer (50 mM Tris-HCl pH 8.0, 500 mM NaCl, 20 mM imidazole, 10%v/v glycerol) and incubated with the supernatant of cell lysate at 4 °C for 1 h. The mixture was loaded onto a Poly-Prep® Chromatography Column, washed 3 times with 5 mL wash buffer, and eluted with 200 μL elution buffer (50 mM Tris-HCl, pH 8.0, 500 mM NaCl, 250 mM imidazole, 10%v/v glycerol) 5 times to obtain purified protein. Purified protein was exchanged to protein buffer (50 mM Tris-HCl, pH 8.0, 150 mM NaCl) using Amicon Ultra column, concentrated to 100 μL, and stored at −20 °C. For western blot detection of His-tagged proteins, the anti-His antibody from Thermo (Catalog number MA121315HRP) was used.

### Biotin labeling of EB3 in crosslinked proteins

Crosslinked proteins were labeled with Biotin Picolyl Azide. Cu^1+^ was prepared using a previously published procedure^37^. The crosslinked proteins were treated with 4 mM Cu^1+^, 4 mM sodium ascorbate, 2 mM 3 [tris(3-hydroxypropyltriazolylmethyl)amine (THTPA), and 500 μM Biotin Picolyl Azide, and the reaction mixture was rotated (800 r.p.m.) at room temperature for 1 h. The CuAAC reaction was terminated by adding 0.2 μL 500 mM EDTA. The buffer of the mixture was exchanged into the protein buffer using Amicon Ultra column.

### Enrichment of crosslinked peptides

Two ways differing in the order of digestion and biotin labeling were used and yielded similar results. Crosslinked proteins were digested by trypsin or GluC at 37 °C overnight in dark, and biotin labeling was then performed on digested products using similar procedures as described above. Alternatively, crosslinked proteins were labeled with biotin first and then digested by trypsin or GluC at 37 °C overnight in dark. Digestion was terminated by adding formic acid at final concentration 5% (v/v). In either case, digested peptides were desalted with Sep-Pak® Vac C18 Cartridges according to manufacturer’s protocol, and eluted peptides were dry down with SpeedVac and resuspended with protein buffer. Streptavidin Agarose slurry 40 μL was rinsed with protein buffer for 3 times and then incubated with resuspended peptides for 1 h at room temperature. Peptide-bound streptavidin beads were washed for 10 times with protein buffer. On-beads, digestion was performed with GluC or trypsin at 37 °C overnight in dark. Beads were then washed with 100 uL 5% formic acid for three times. All wash fractions were combined and desalted with homemade C18 StageTips. For western blot analysis of biotin labeling, streptavidin-HRP conjugate from Bio-Rad (Catalog number 1610381) was used.

### General mass spectrometric analysis

Peptides were separated by EASY-Spray PepMap C18 Columns (50 cm; particle size, 2 μm; pore size, 100 Å; Thermo Fisher) using an in-line EASY-spray source and nano-LC UltiMate 3000 high-performance liquid chromatography system (Thermo Fisher). Peptides were eluted over a linear gradient from 3 to 40% solvent B for 60 min at a flow rate of 300 nL min^−1^ (mobile phase A: 2% acetonitrile, 98% H_2_O, 0.1% formic acid; mobile phase B: 80% acetonitrile, 20% H_2_O, 0.1% formic acid). MS/MS data were collected in a data-dependent manner using a top 10 method with a full MS mass range from 400 to 1600 *m/z*, 60,000 resolution, and an AGC target of 1 × 10^6^. MS2 scans were triggered when an ion intensity threshold of 5 × 10^4^ was reached, and peptides were fragmented in ion trap. A dynamic exclusion time of 30 s was used, and singly charged ions were excluded.

### In vitro ubiquitination assay

Reactions containing 20 μM UBE2D3, 20 μM yPCNA, 50 nM E1, and 200 μM ubiquitin in buffer containing 50 mM Tris pH 8.0, 50 mM NaCl, 10 mM MgCl_2_ and 10 mM ATP were run at 37 °C for 1 h. Reactions were terminated by SDS-PAGE loading buffer and analyzed by SDS-PAGE gel and Western blot.

### Azi- or Bpa-mediated photo-crosslinking of proteins

Affibody_8C_His6 and MBP-Z_24TAG_His6 together with tRNATyr CUA/AziRS for Azi (or tRNATyr CUA/BpaRS for Bpa) were expressed in *E. coli* BL21 cells in the presence of 1 mM Azi (or Bpa). Cells were incubated for 6 h at 30 °C after induction with arabinose and IPTG at final concentration of 0.2% and 0.5 mM, respectively. Cells were pelleted and resuspended in PBS buffer, followed by irradiation with a handheld UV lamp (365 nm, 8 W) for 30 min. One aliquot of cells was boiled with SDS-PAGE loading buffer and analyzed with western blot; others cells were used for His-tag purification of cross-linked proteins, which were subsequently analyzed with SDS-PAGE gel and western blot.

### Small molecular chemical cross-linking

In a 20-μL reaction (20 mM Hepes, pH 7.5, 150 mM NaCl), 20 μM UBE2D3 and 20 μM yPCNA were cross-linked at room temperature for 1 h with BS^3^ at 2 mM, which corresponded to a 1:100 protein:cross-linker molar ratio, using protocols previously published^30^. Reaction was terminated with 20 mM ammonium bicarbonate for 20 min at room temperature. Control samples of yPCNA and UBE2D3 were also separately cross-linked with the same experimental conditions.

### High-resolution tandem mass spectrometric analysis

Digested peptides were loaded onto pre-column (100 μm × 4 cm, 3 μm C18) and analyzed by analytical column (75 μm × 1 cm, 1.8 μm C18, 5 μm tip) with EASY-nLC 1000 system. Samples were analyzed with a 60 min linear gradient at flow rate 200 nL min^−1^ as follows: 0–5% B for 2 min, 5–40% B for 29 min, 40–60% B in 15 min, 60–100% B in 5 min, 100% B for 10 min. The Q-Exactive mass spectrometer was operated in data-dependent mode with one full MS scan at *R* = 70,000 (*m/z* = 200), followed by ten HCD MS/MS scans at *R* = 17,500 (*m/z* = 200), NCE = 27, with an isolation width of 2 m/z. The AGC targets for the MS1 and MS2 scans were 3 × 10^6^ and 1 × 10^5^, respectively, and the maximum injection time for MS1 and MS2 were both 60 ms. Precursors of the + 1, + 7 or above, or unassigned charge states were rejected; exclusion of isotopes was disabled; dynamic exclusion was set to 30 s. Mass spectrometry data were searched by Maxquant.

### Data availability

The MS data that support the findings of this study are available from http://www.huanglab.org.cn/yangbing/NatComm.

## Electronic supplementary material

Supplementary Information
