# Supplementary material for: Spontaneous and specific chemical cross-linking in live cells to capture and identify protein interactions
Source: Nat Commun. 2017 Dec 21;8:2240. doi: 10.1038/s41467-017-02409-z (PMC5740110; doi:10.1038/s41467-017-02409-z)
Supplement: Supplementary file 1 — Supplementary Information [file 41467_2017_2409_MOESM1_ESM.pdf]

Spontaneous and specific chemical cross-linking in live cells to capture and identify protein interactions

Bing Yang[1,5], Shibing Tang[2,5], Cheng Ma[3,5], Shang-Tong Li[4], Guang-Can Shao[4], Bobo Dang[1], William F. DeGrado[1], Meng-Qiu Dong[4], Peng George Wang[3], Sheng Ding[2], Lei Wang[1*]

1. Department of Pharmaceutical Chemistry and the Cardiovascular Research Institute, University of California San Francisco, 555 Mission Bay Blvd. South, San Francisco, California, 94158 USA
2. Gladstone Institute of Cardiovascular Disease and Department of Pharmaceutical Chemistry, University of California San Francisco, 1650 Owens St., San Francisco, California, 94158 USA
3. Department of Chemistry and Center for Therapeutics and Diagnostics, Georgia State University, P.O. Box 3965, Atlanta, Georgia, 30302 USA
4. National Institute of Biological Sciences, 7 Science Park Rd., Beijing, 102206, China
5. These authors contributed equally to this work

Correspondence should be addressed to L.W. (Lei.Wang2@ucsf.edu)

Supplementary Methods

Synthesis of Uaa EB3.

General information. All commercially available reagents were purchased from Sigma-Aldrich and used without further purification. Solvents (ACS grade) were purchased from Sigma-Aldrich or Fisher Scientific and used without further purification unless otherwise noted. Tetrahydrofuran (THF) was dried by standard methods prior to use. Oxygen- and moisture-sensitive reactions were carried out under argon atmosphere. Reactions were monitored by thin-layer chromatography (TLC) carried out on 0.25 mm EMD Millipore TLC Silica Gel 60 F_{254} using UV light for visualization and an ethanolic solution of phosphomolybdic acid under heat or powdered iodine for developing. Flash column chromatography was generally performed on silica gel (200-300 mesh). Yields refer to chromatographically homogeneous materials. High-resolution mass spectra (HRMS) were obtained using electrospray ionization (ESI). The ^{1}H and ^{13}C NMR spectra were recorded on a Varian 400 spectrometer at 400 MHz and 100 MHz, respectively. The chemical shifts (*δ*) were calibrated using residual undeuterated solvent as an internal reference and reported in ppm and coupling constants (*J*) in Hz. The following abbreviations were used to explain the multiplicities: s = singlet, d = doublet, t = triplet, q = quartet, p = quintet, m = multiplet.

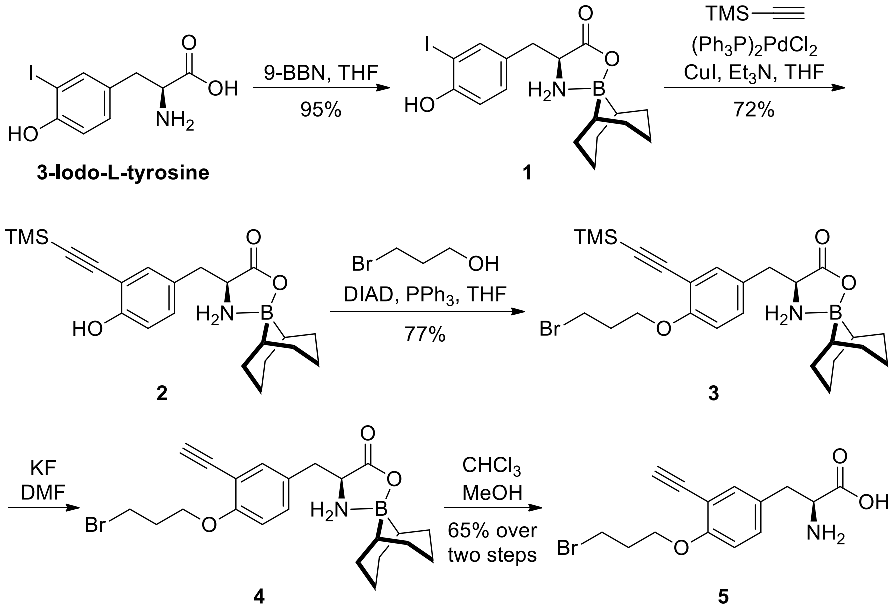

Overall scheme for synthesis of Uaa EB3 (5). 3-Iodo-L-tyrosine was protected with 9-BBN to give compound **1** that was coupled with ethynyltrimethylsilane under the Sonogashira cross-coupling conditions to yield compound **2**. Compound **3** then was synthesized by Mitsunobu reaction, which gives the final Uaa **5** after deprotection of alkyne and release of free amino acid.

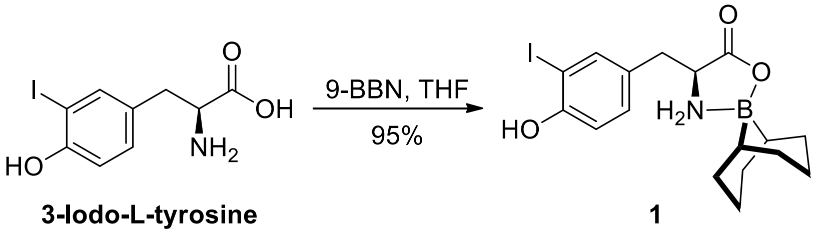

3-Iodo-L-tyrosine **1**

Compound 1. Compound 1 was synthesized from commercially available 3-iodo-L-tyrosine in 95% yield as a white powder by a reported protocol, and the spectral data of which were in good agreement with those reproted[1, 2]. ^{1}H NMR (400 MHz, CD_3OD) δ 7.70 (d, J = 1.6 Hz, 1H), 7.15 (dd, J = 1.6, 8.0 Hz, 1H), 6.81 (d, J = 8.0 Hz, 1H), 3.90 (dd, J = 5.2, 7.6 Hz, 1H), 3.16 (dd, J = 5.2, 14.8 Hz, 1H), 2.96 (dd, J = 7.6, 14.8 Hz, 1H), 1.83-1.44 (m, 12H), 0.54 (s, 1H), 0.23 (s, 1H).

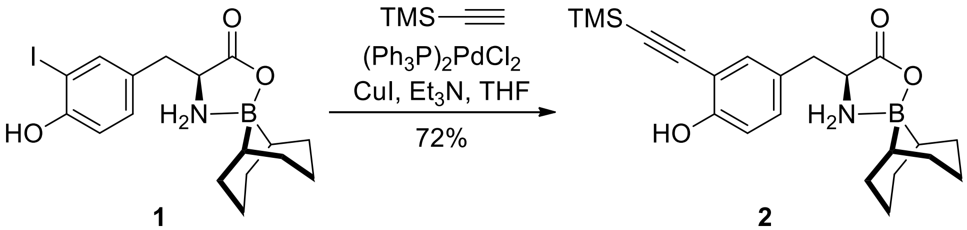

1 **2**

Compound 2. Compound 2 was synthesized from compound 1 in 72% yield as a light yellow solid by a reported protocol, and the spectral data of which were in good agreement with those reported[1, 2]. ^{1}H NMR (400 MHz, CD_3OD) δ 7.31 (d, J = 2.0 Hz, 1H), 7.14 (dd, J = 2.0, 8.4 Hz, 1H), 6.82 (d, J = 8.4 Hz, 1H), 6.41 (dd, J = 8.0, 11.2 Hz, 1H), 5.14 (dd, J = 8.0, 11.2 Hz, 1H), 3.91 (m, 1H), 3.16 (dd, J = 5.2, 14.8 Hz, 1H), 2.97 (dd, J = 8.0, 14.8 Hz, 1H), 1.86-1.43 (m, 12H), 0.55 (s, 1H), 0.28 (s, 1H), 0.23 (s, 9H).

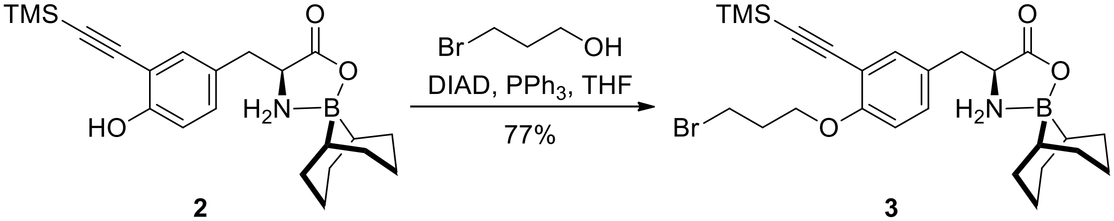

2 **3**

Compound 3. To a mixture of compound 2 (1.192 g, 3.0 mmol), 3-bromo-1-propanol (834 mg, 6.0 mmol), triphenylphosphine (1.574 g, 6.0 mmol) in 30 mL dry THF, diethyl azodicarboxylate (DIAD, 1.20 mL, 6.0 mmol) was added drop-wise with stirring at 0 ^{o}C. The reaction was slowly warmed to room temperature and stirred for additional 2 h. The solvent was removed under reduced pressure, and the residue was purified by flash column chromatography (hexanes/ethyl acetate, 50/50, v/v) to afford the compound 3 in 77% yield (1.201g) as a light yellow solid. ^{1}H

NMR (400 MHz, $CDCl_3$) δ 7.29 (d, J = 2.0 Hz, 1H), 7.17 (dd, J = 2.0, 8.4 Hz, 1H), 6.88 (d, J = 8.4 Hz, 1H), 4.97 (t, J = 9.2 Hz, 1H), 4.15 (t, J = 5.6 Hz, 2H), 3.98 (p, J = 4.4 Hz, 1H), 3.87 (dd, J = 6.0, 10.8 Hz, 1H), 3.68 (t, J = 6.4 Hz, 2H), 3.29 (dd, J = 4.4, 14.8 Hz, 1H), 3.01 (dd, J = 5.2, 14.8 Hz, 1H), 2.35 (p, J = 6.0 Hz, 2H), 1.82-1.32 (m, 12H), 0.57 (s, 1H), 0.36 (s, 1H), 0.24 (s, 9H); ^{13}C NMR (100 MHz, $CDCl_3$) δ 172.8, 159.4, 134.0, 130.7, 126.8, 114.0, 113.3, 100.2, 100.1, 66.1, 56.4, 35.5, 32.3, 31.6, 31.1, 29.7, 24.3, 23.7, 0.1; HRMS Calcd. for $C_{25}H_{38}BBrNO_3Si$ $[M+H]^+$ 518.1892, found 518.1885.

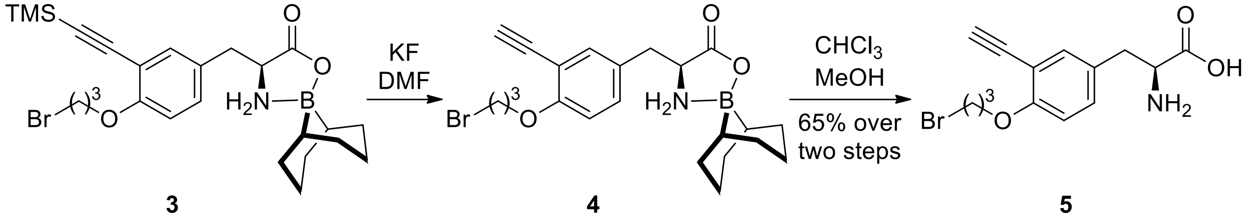

3 4 5

Compound 4 and compound 5. To a solution of compound 3 (1.201g, 2.32 mmol) in 8.0 mL wet DMF, KF (134.8 mg, 2.32 mmol) was added with stirring. The reaction was stirred for additional 2 h at ambient temperature, then was quenched with 1 M $KHSO_4$ (8.0 mL). The solution was extracted with EtOAc (3 × 30 mL). The combined EtOAc layers were washed with water (3 × 10 mL) and brine (10 mL) then dried over Na_2SO_4, filtered, and concentrated to provide crude compound 4 that was used as such in the next step.

The above obtained compound 4 was dissolved in methanol (2 mL) and diluted with chloroform (20 mL). The solution was stirred at room temperature for 24 h. Hexanes (60 mL) was added to the solution, which led to precipitation of the compound 5. The crude product was washed with dichloromethane (2 × 3 mL) and diethyl ether (2 × 6 mL) and was dried in vacuo to afford final compound 5 in 65% yield over two steps (491.9 mg) as a white powder. ^1H NMR (400 MHz, CD_3OD) δ 7.38 (d, J = 2.4 Hz, 1H), 7.27 (dd, J = 2.4, 8.4 Hz, 1H), 7.01 (d, J = 8.4 Hz, 1H), 4.18 (t, J = 5.2 Hz, 2H), 3.40 (t, J = 5.6 Hz, 1H), 4.00 (t, J = 5.6 Hz, 1H), 3.68 (t, J = 6.0 Hz, 2H), 3.63 (s, 1H), 3.22 (dd, J = 4.4, 14.4 Hz, 1H), 3.04 (dd, J = 8.0, 14.4 Hz, 1H), 2.31 (p, J = 6.0 Hz, 2H); ^{13}C NMR (100 MHz, CD_3OD) δ 173.3, 160.6, 135.8, 132.3, 129.2, 114.0, 113.8, 82.7, 80.6, 67.3, 57.1, 37.0, 33.5, 30.6; HRMS Calcd. for $C_{14}H_{17}BrNO_3$ $[M+H]^+$ 326.0386, found 326.0389.

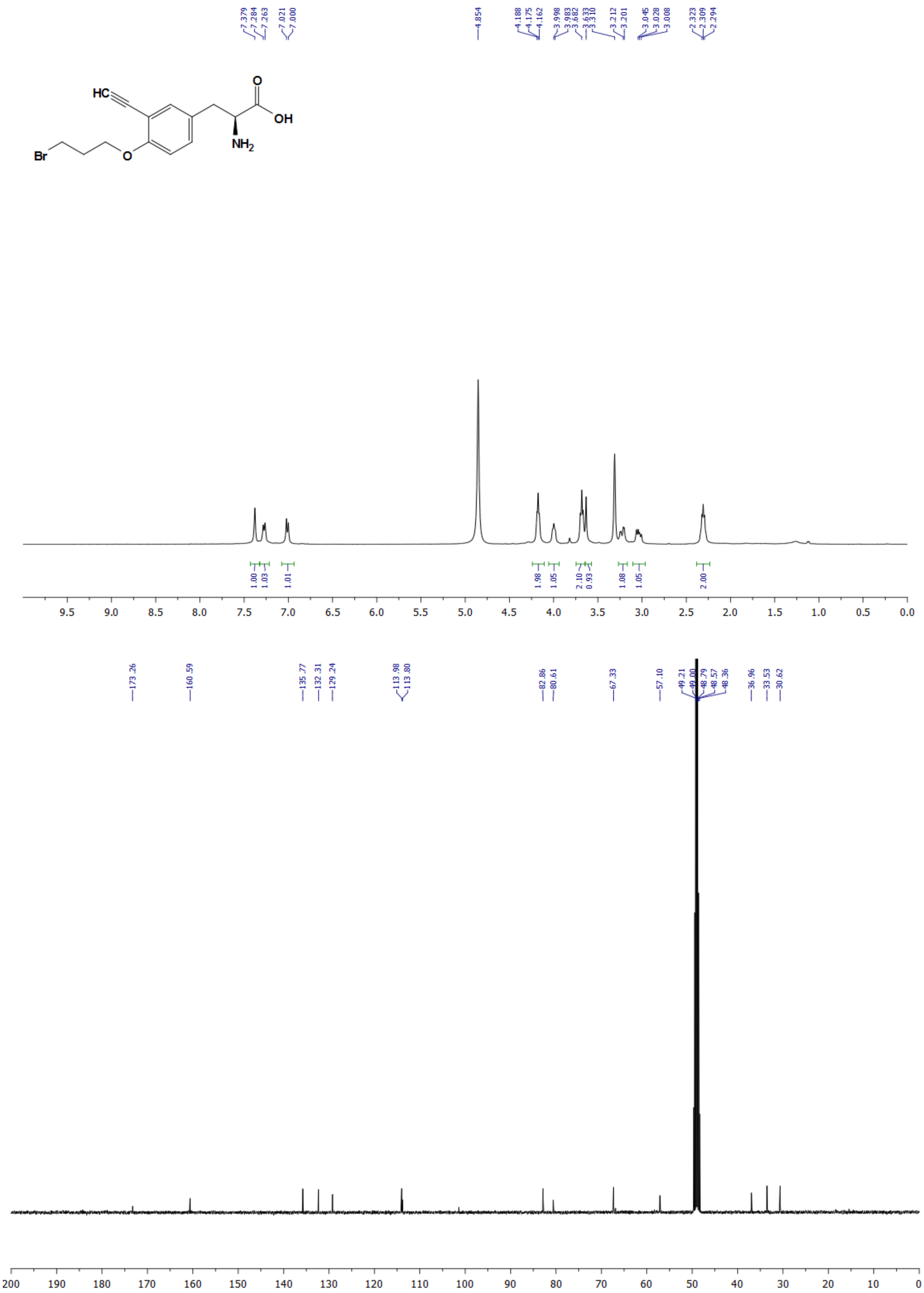

Supplementary Figure 1. ^{1}H NMR (400 MHz, top) and ^{13}C NMR (100 MHz, bottom) spectra of UAA EB3.

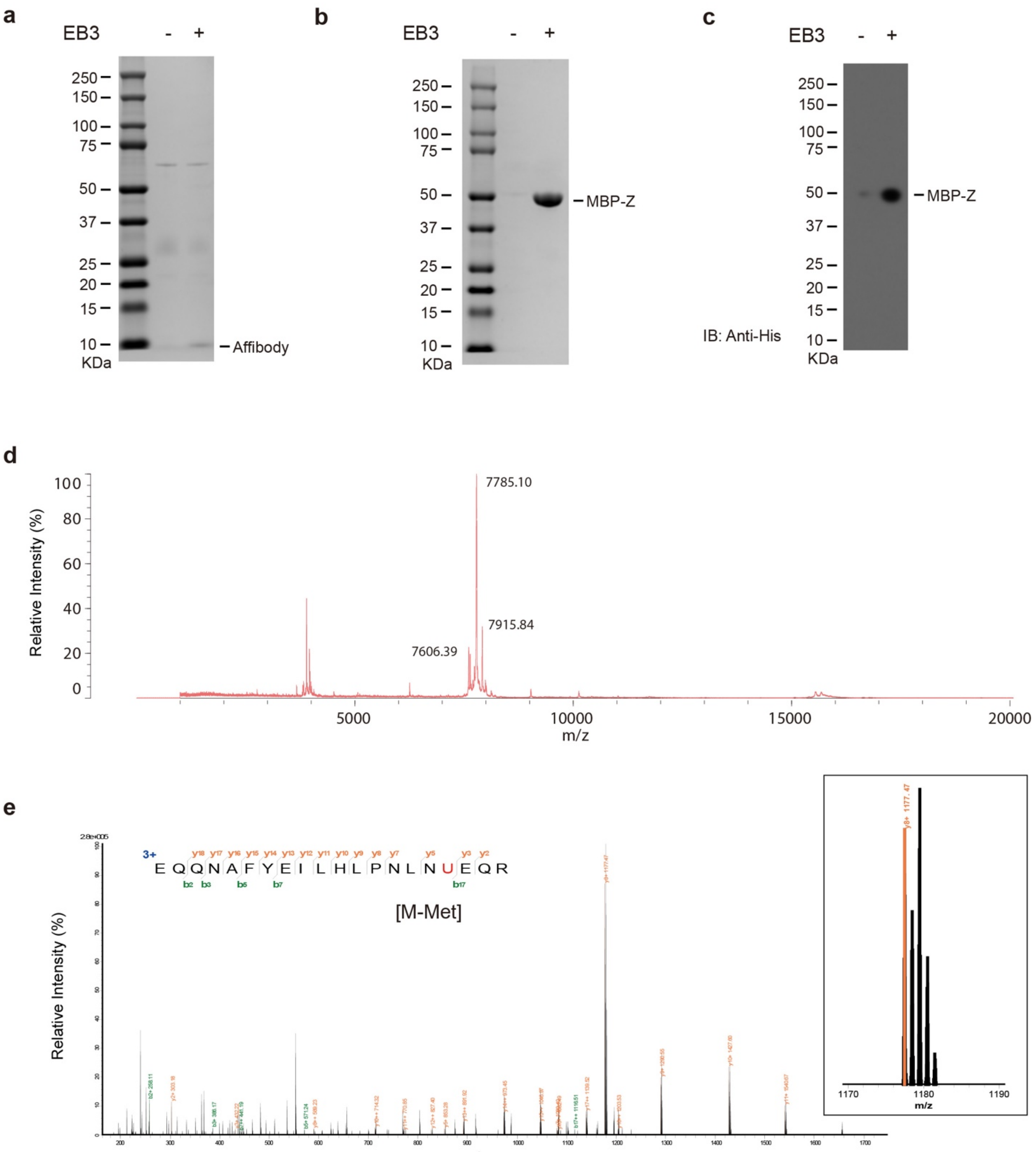

Supplementary Figure 2. Genetic incorporation of EB3 into proteins in *E. coli*. (**a**) SDS-PAGE gel analysis of affibody(36TAG) expression in *E. coli* in the absence and presence of EB3. (**b**) SDS-PAGE gel of expression of MBP-Z(24TAG) in *E. coli* in the absence and presence of EB3. (**c**) Western blot analysis of MBP-Z(24TAG) expression in *E. coli* in the absence and presence of EB3. (**d**) MALDI-TOF analysis of EB3-incorporated intact affibody. Expected mass: $[M]^+$ = 7913.91 Mono.; 7919.61 Avg. $[M\text{-}Met]^+$ = 7782.87 Mono.; 7788.42 Avg. (**e**) Tandem mass spectrum of EB3-incorporated MBP-Z peptide. U indicates EB3. The insert is the zoomed-in peak for y8 ion, which showed correct bromine isotope pattern. This distinct bromine isotope pattern is present in all other peaks for Uaa-containing ions, further confirming the incorporation of EB3.

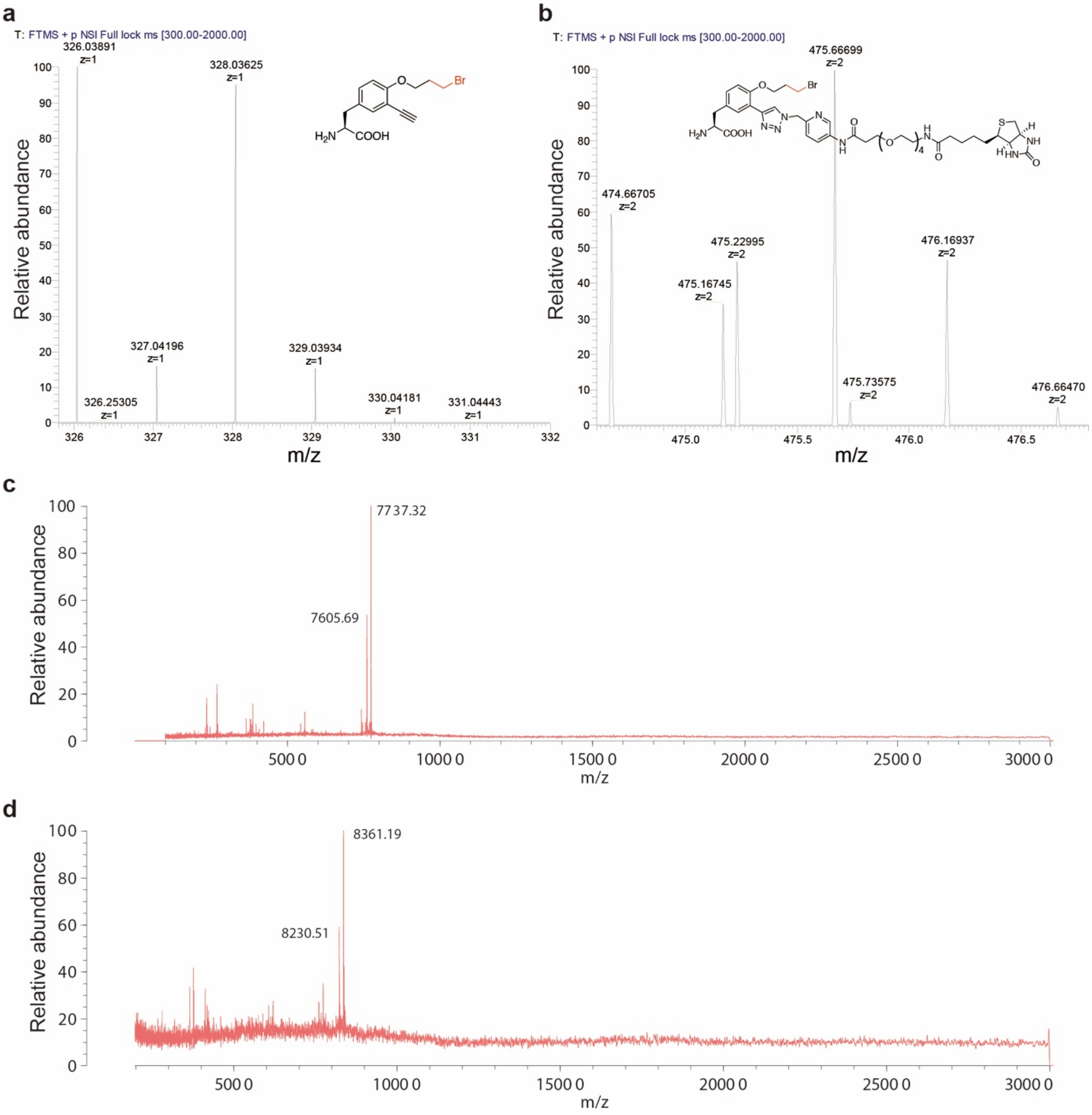

Supplementary Figure 3. Labeling of biotin onto EB3 via CuAAC at the amino acid and protein level. (**a**) High resolution mass spectrum of EB3 (Expected mass: $[M+H]^+$ = 326.03863 mono). (**b**) High resolution mass spectrum of biotin labeled EB3 (Expected mass: $[M+2H]^{2+}$ = 474.66808 mono) (**c**) MALDI-TOF spectrum of EB3 incorporated affibody (Expected mass: $[M+H]^+$ = 7731.43 Avg, $[M+H\text{-}Met]^+$ = 7600.24 Avg.) (**d**) MALDI-TOF spectrum of biotin labeled, EB3 incorporated affibody (Expected mass: $[M+H]^+$ = 8354.17 Avg, $[M+H\text{-}Met]^+$ = 8222.97 Avg.)

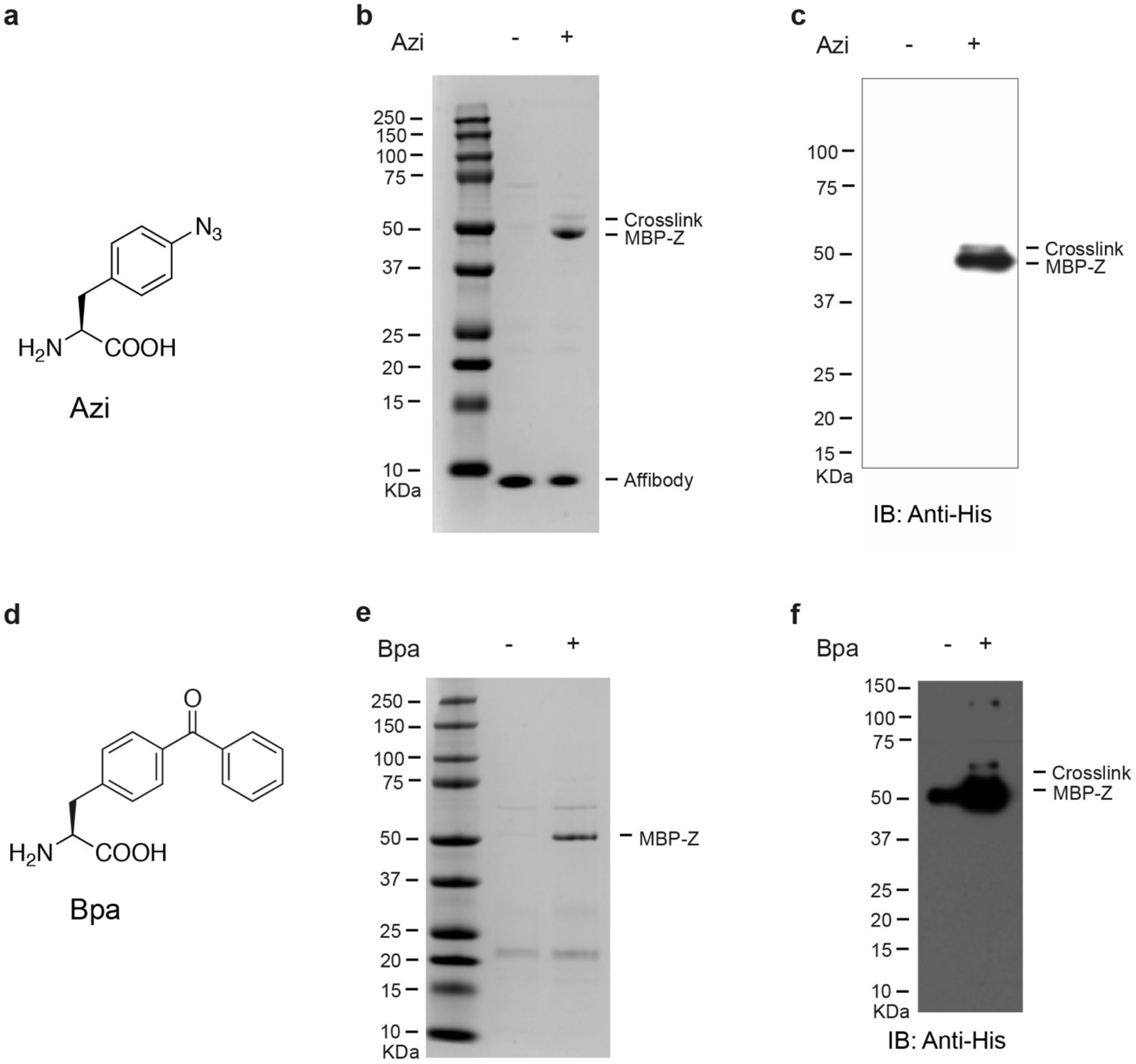

Supplementary Figure 4. Photo-crosslinking of affibody to Z protein in *E. coli* via the genetically encoded Azi or Bpa. (**a**) Structure of the photo-crosslinking Uaa Azi. (**b**) SDS-PAGE gel of His-tag purified proteins from cells expressing affibody(7C)_His6 and MBP-Z(24Azi)_His6 that had been photo-crosslinked by UV light. (**c**) Western blot analysis of cell lysate of cells expressing affibody(7C)_His6 and MBP-Z(24Azi)_His6 that had been photo-crosslinked by UV light. (**d**) Structure of the photo-crosslinking Uaa Bpa. (**e**) SDS-PAGE gel of His-tag purified proteins from cells expressing affibody(7C)_His6 and MBP-Z(24Bpa)_His6 that had been photo-crosslinked by UV light. (**f**) Western blot analysis of cell lysate of cells expressing affibody(7C)_His6 and MBP-Z(24Bpa)_His6 that had been photo-crosslinked by UV light.

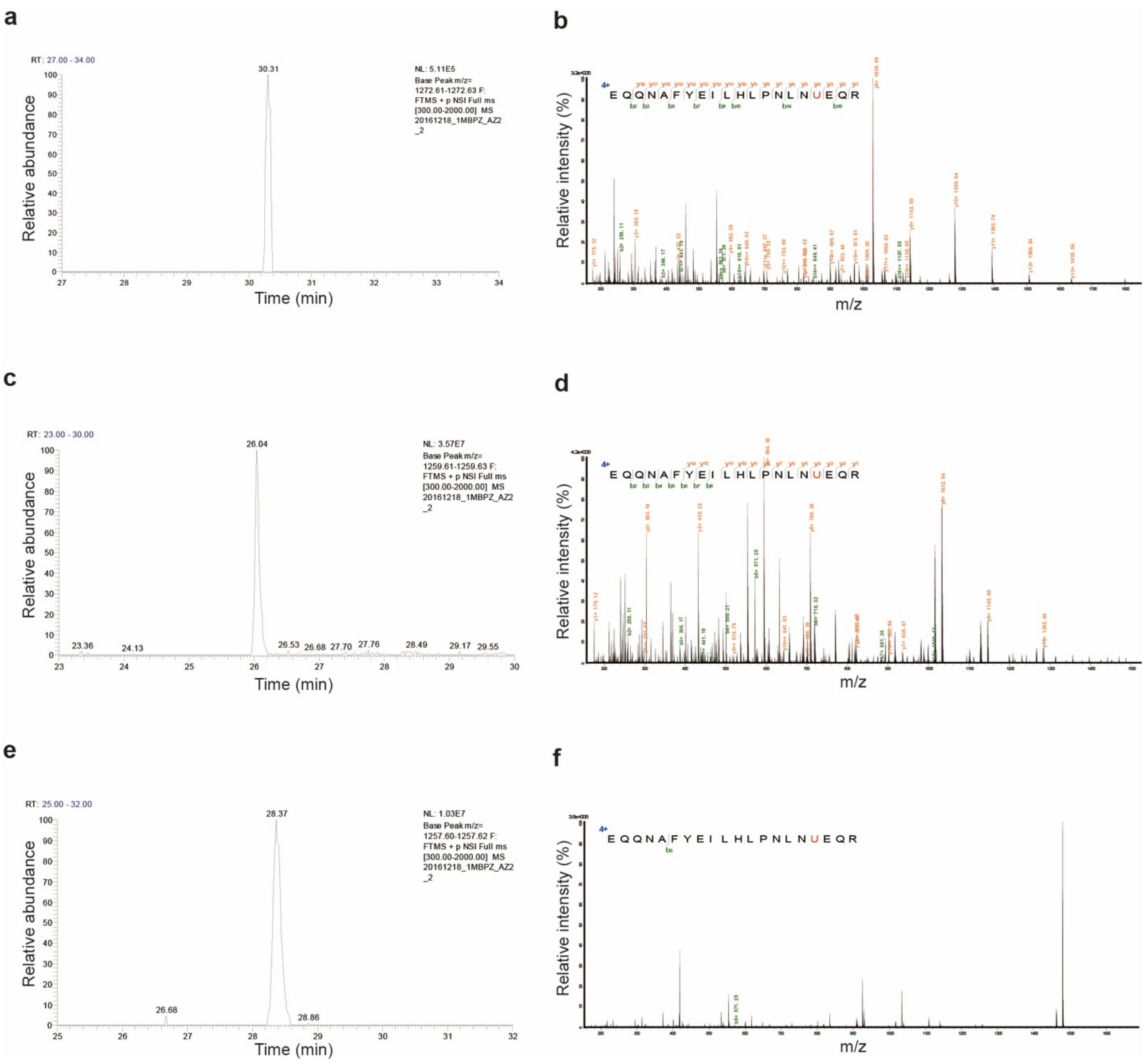

Supplementary Figure 5. Products of Azi-incorporated peptide after photo-crosslinking. (**a**) Extracted ion chromatography of Azi-incorporated peptides. (**b**) Tandem mass spectrum of Azi-incorporated peptides. (**c**) Extracted ion chromatography of Azi-incorporated peptides (-2N+2H). (**d**) Tandem mass spectrum of Azi-incorporated peptides (-2N+2H). (**e**) Extracted ion chromatography of Azi-incorporated peptides (-2N-2H). (**f**) Tandem mass spectrum of Azi-incorporated peptides (-2N-2H). U indicates the Azi incorporation site.

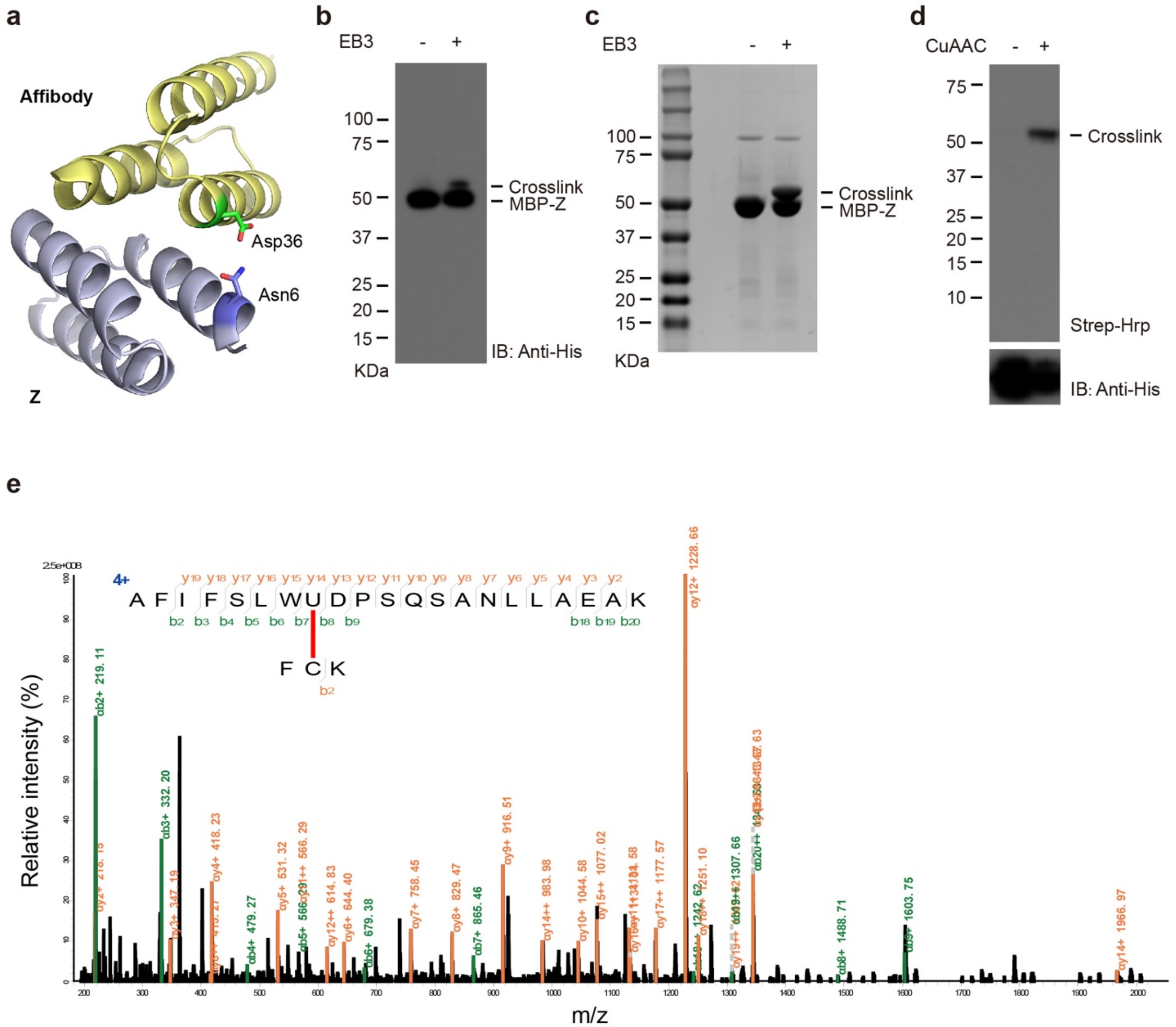

Supplementary Figure 6. Validation of *in vivo* cross-linking of affibody(36EB3) with MBP-Z(6C) and biotin labeling of the crosslinked complex. (**a**) Structure of the affibody-Z protein complex (PDB ID 1LP1), with two proximal sites Asp36 in the affibody and Asn6 in the Z protein highlighted. (**b**) Western blot of cell lysate of cells expressing affibody(36EB3) and MBP-Z(6C). (**c**) SDS-PAGE gel of His-tag purified proteins from cells expressing affibody(36EB3) and MBP-Z(6C). (**d**) Western blot of cross-linked affibody(36EB3) and MBP-Z(6C) before and after biotin labeling via CuAAC click reaction. (**e**) Mass spectrum of cross-linked peptide between affibody(36EB3) and MBP-Z(6C). The crosslink was clearly mapped to EB3 at site 36 of the affibody and the Cys6 of the Z protein. U represents EB3 in the peptide sequence.

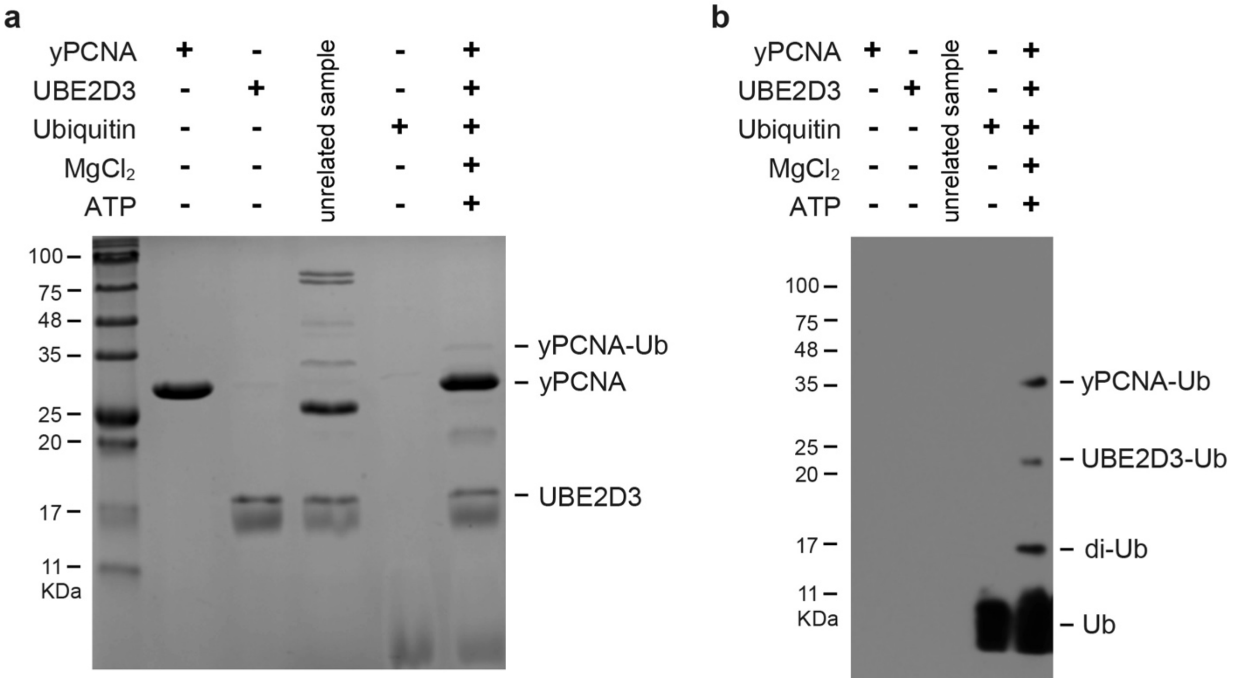

Supplementary Figure 7. *In vitro* ubiquitination assay showing that UBE2D3 ubiquitinates yPCNA. (**a**) SDS-PAGE gel of *in vitro* ubiquitination assay. (**b**) Western blot analysis of *in vitro* ubiquitination assay. A Flag tag was appended at the N-terminus of Ubiquitin, and was detected with an anti-Flag antibody (Catalog number F9291, Sigma-Aldrich).

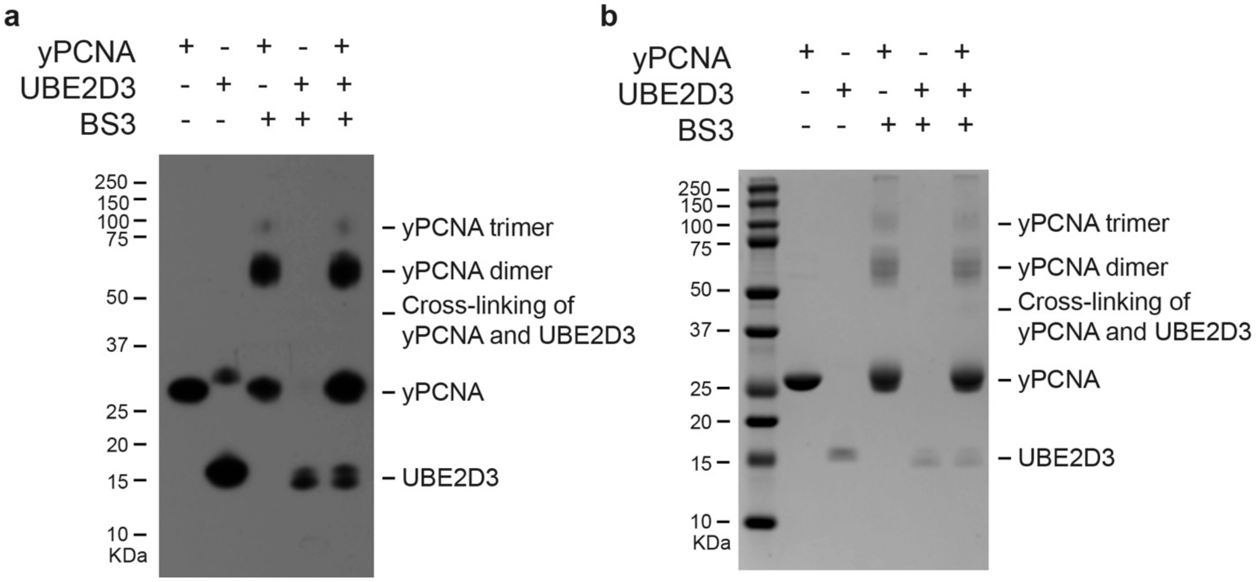

Supplementary Figure 8. *In vitro* crosslinking of yPCNA with UBE2D3 using small molecule chemical cross-linker BS3 yielded no crosslinking. (**a**) Western blot analysis of BS3 crosslinking yPCNA and UBE2D3. (**b**) SDS-PAGE gel of BS3 crosslinking of yPCNA and UBE2D3. No crosslinking band for yPCNA/UBE2D3 complex was detected in either SDS-PAGE or Western blot.

a

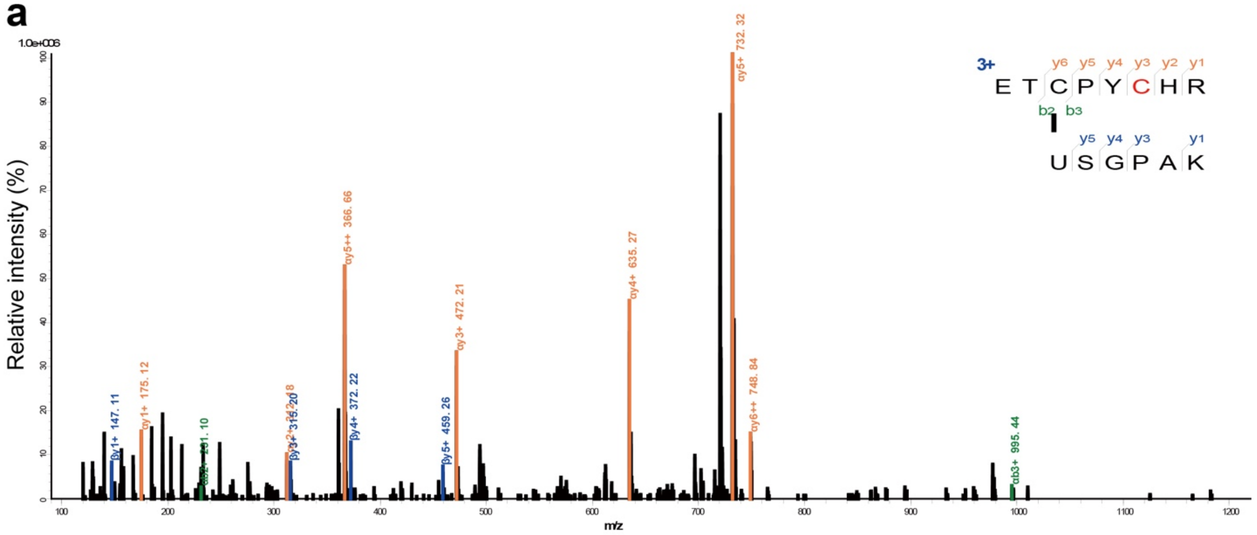
1.0e+006
Relative intensity (%)
m/z
3+
ETCPYCHR
y6 y5 y4 y3 y2 y1
b2 b3
USGPAK
y5 y4 y3 y1
αy5+ 732.32
αy5++ 366.66
αy4+ 635.27
αy3+ 472.21
αy1+ 175.12
αy6++ 748.84
βy1+ 147.11
βy3+ 315.20
βy4+ 372.22
βy5+ 459.26
αb3+ 995.44

b

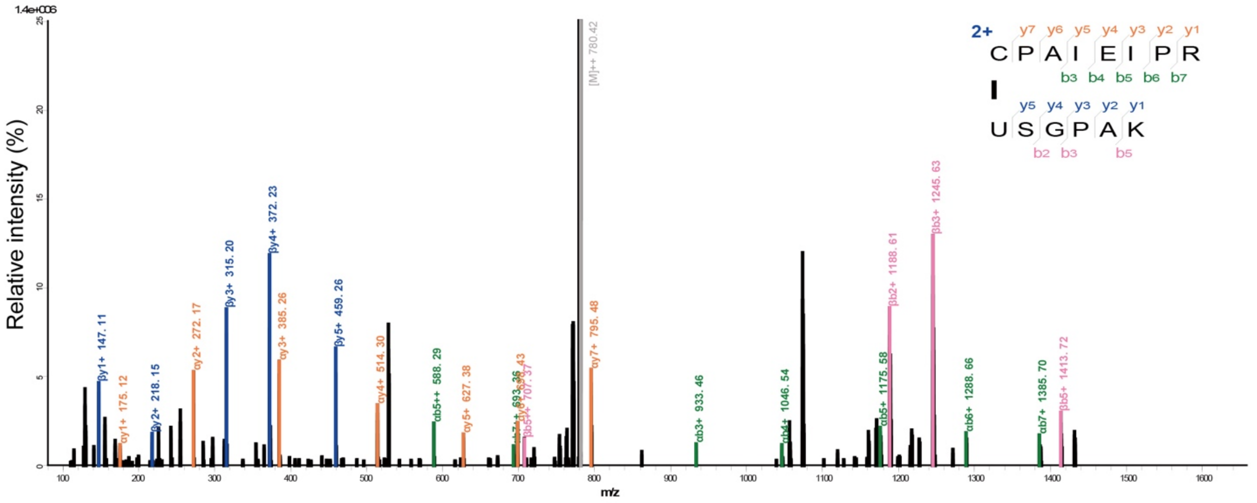
1.4e+006
Relative intensity (%)
m/z
2+
CPAIEIPR
y7 y6 y5 y4 y3 y2 y1
b3 b4 b5 b6 b7
USGPAK
y5 y4 y3 y2 y1
b2 b3 b5
[M]++ 780.42
βy1+ 147.11
αy1+ 175.12
βy2+ 218.15
αy2+ 272.17
βy3+ 315.20
βy4+ 372.23
αy3+ 385.26
βy5+ 459.26
αy4+ 514.30
αb5++ 588.29
αy5+ 627.38
αy7+ 795.48
αb3+ 933.46
αb4+ 1046.54
αb5+ 1175.58
βb2+ 1188.61
βb3+ 1245.63
αb6+ 1288.66
αb7+ 1385.70
βb5+ 1413.72

c

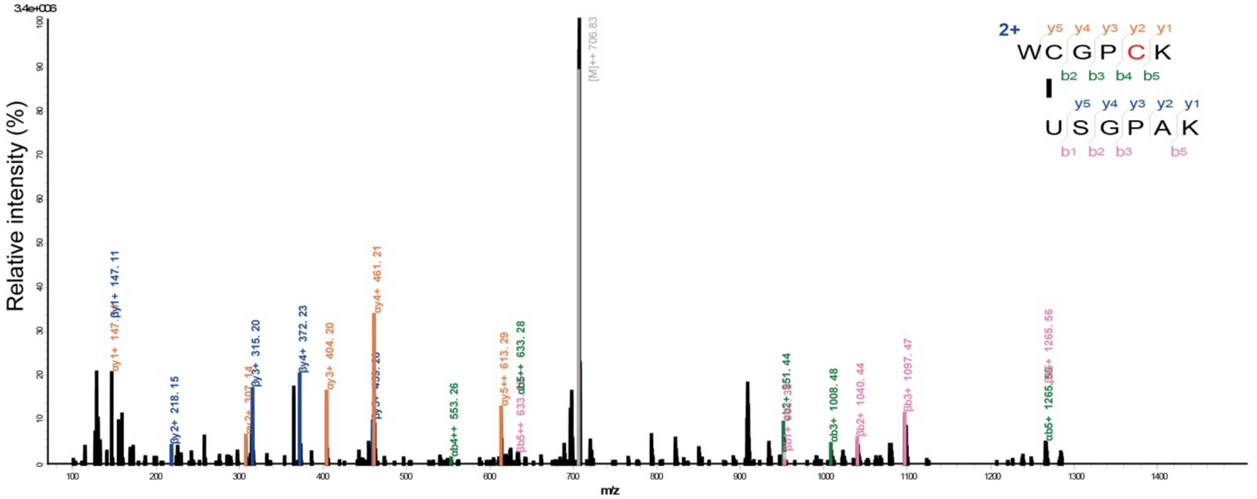
3.4e+006
Relative intensity (%)
m/z
2+
WCGPCK
y5 y4 y3 y2 y1
b2 b3 b4 b5
USGPAK
y5 y4 y3 y2 y1
b1 b2 b3 b5
[M]++ 706.83
βy1+ 147.11
βy2+ 218.15
βy3+ 315.20
βy4+ 372.23
αy3+ 404.20
αy4+ 461.21
αb4++ 553.26
αy5++ 613.29
αb5++ 633.28
αb3+ 1008.48
βb2+ 1040.44
βb3+ 1097.47
αb5+ 1265.56

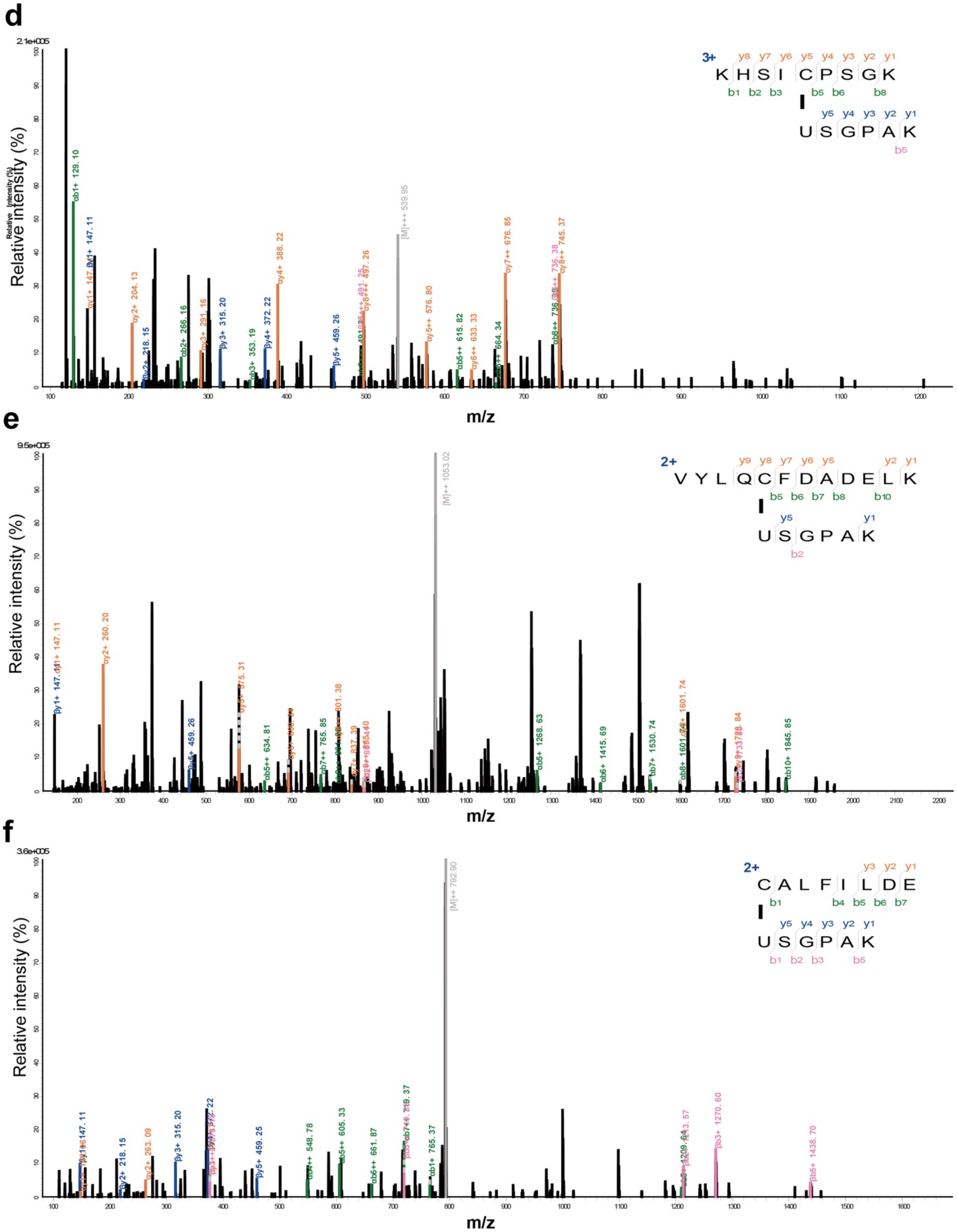

Supplementary Figure 9. Mass spectrometric analysis of *in vivo* crosslinking of Trx1(U32-S33-A36) to endogenous proteins in *E. coli* cells. Tandem mass spectra are shown for Trx1 crosslinked with GLRX3 (**a**), yccU (**b**), trxA (**c**), ppiC (**d**), glpQ (**e**), and dhaR (**f**). U represents

BprY in the peptide sequence. The monoisotopic peak of the precursor ion is labeled in grey. In **e**, the unlabeled major peaks came from a peptide acetyl-SDKIIHLTDDSFDTDVLK ($[M+2H]^{2+}$ = 1052.527), which was co-eluted with the crosslinked peptide (VYLQCFDADELK-USPGAK, $[M+2H]^{2+}$ = 1053.017) with similar retention time and very close m/z, as shown in Supplementary Figure 10.

a

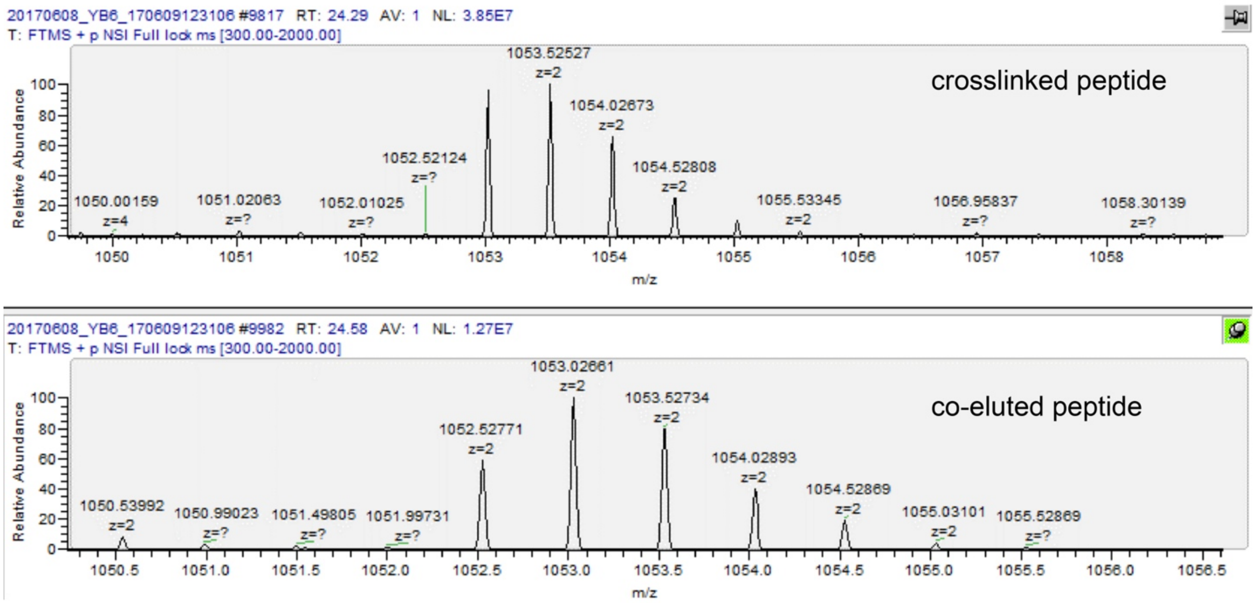

b

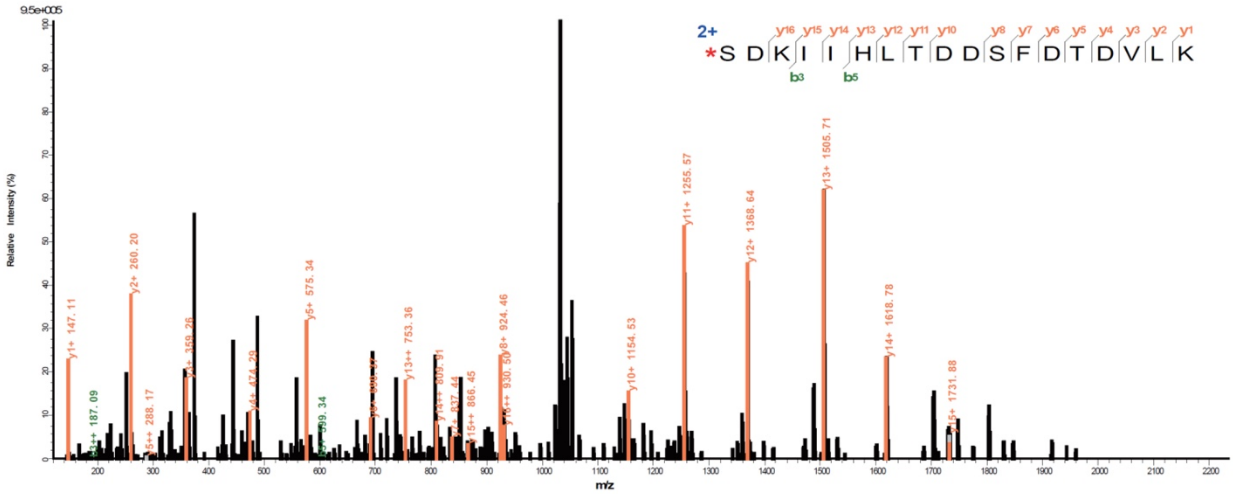

Supplementary Figure 10. A peptide co-eluted with the crosslinked peptide in Supplementary Figure 9e. (**a**) Precursor ion for the crosslinked peptide (VYLQCFDADELK-USPGAK, $[M+2H]^{2+}$ = 1053.017) and the co-eluted peptide (acetyl-SDKIIHLTDDSFDTDVLK, $[M+2H]^{2+}$ = 1052.527). (**b**) Tandem mass spectrum for the co-eluted peptide acetyl-SDKIIHLTDDSFDTDVLK.

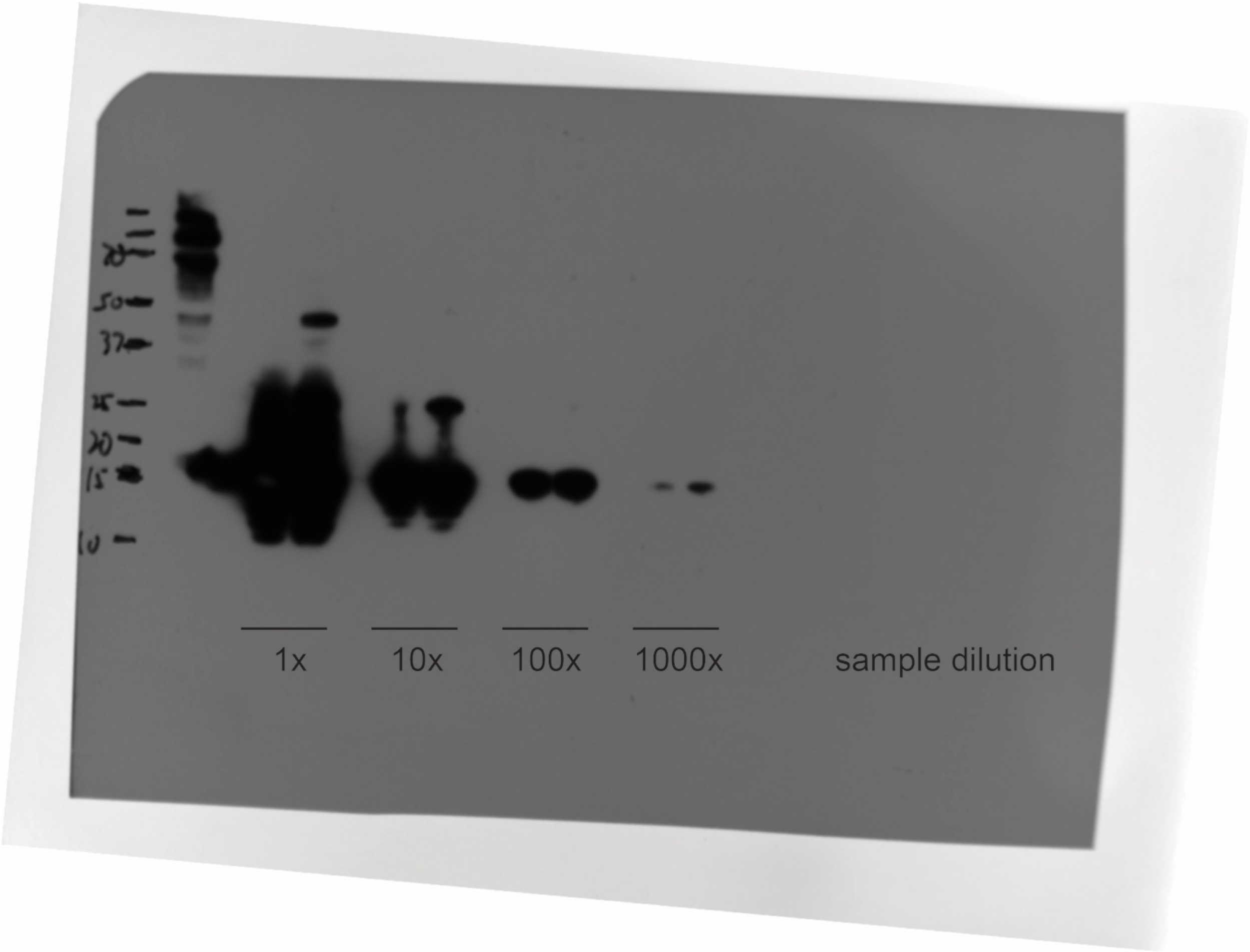

Supplementary Figure 11. Full Western blot of Figure 3c.

Supplementary Table 1. Thioredoxin-interacting proteins identified by GECX-MS.

Majority protein IDs	Fasta headers	#Peptides	Sequence coverage [%]	Mol. weight [kDa]	#Cys	Gene Name
A0A0A7L5L4	L-glutamine:D-fructose-6-phosphate aminotransferase	47	62.9	66.894	4	glmS
A0A0A7L5N5	Alkyl hydroperoxide reductase, F52a subunit, FAD/NAD(P)-binding	25	45.9	56.176	6	ahpF
A0A0A7KXP5	Catalase HPII, heme d-containing	24	36.3	84.162	2	katE[3]
A0A0A7L6I2	DNA-binding transcriptional dual regulator	21	74.8	23.64	3	crp
A0A0A7L310	GTP cyclohydrolase I	16	61.3	24.83	2	folE
A0A0A7L6H5	FKBP-type peptidyl prolyl cis-trans isomerase (Rotamase)	15	59.7	20.853	6	slyD[4]
A0A0A7L4R7	Adenylosuccinate synthetase	14	39.6	47.344	4	purA[5]
A0A0A7L4A9	Alpha-galactosidase, NAD(P)-binding	13	35	50.657	12	melA
A0A0A7KX67	Uridylyltransferase	13	15.6	102.39	9	glnD
P39451	Alcohol dehydrogenase, propanol-preferring	13	49.1	35.379	9	adhP[6]
A0A0A7KXZ0	Fused ribonucleaseE: endoribonuclease/RNA-binding protein/RNA degradosome binding protein	13	13.6	118.2	4	rne
A0A0A7KYQ0	16S rRNA pseudouridine(516) synthase	13	59.7	25.865	2	rsuA
B8LFD6	Beta-galactosidase	12	13.2	116.48	16	lacZ
A0A0A7L5T7	Thioredoxin 1	12	73.4	11.806	2	trxA[7]

A0A0A7L4G9	tRNA s(4)U8 sulfurtransferase	11	20.5	54.972	5	thiI
A0A0A7L661	Protein chain elongation factor EF-Tu (Duplicate of tufA)	11	34	43.313	3	tufB[4]
A0A0A7L440	Glycerol dehydrogenase, NAD	10	30	38.712	7	gldA
A0A0A7L0K3	Fused histidinol-phosphatase/imidazoleglycerol-phosphate dehydratase	10	31	40.278	6	hisB
A0A0A7L1V2	Succinylornithine transaminase, PLP-dependent	10	24.1	43.665	6	astC
A0A0A7KYL9	Putative DNA-binding transcriptional regulator	10	41	32.723	3	yeiE
A0A0A7L9M9	dTDP-4-deoxyrhamnose-3,5-epimerase	10	50.8	21.27	3	rfbC
A0A0A7L3M4	Catalase-peroxidase HPI, heme b-containing	10	14.6	80.023	1	katG[7]
A0A0A7L1F0	Fumarate hydratase (Fumarase A), aerobic Class I	9	19.5	60.298	9	fumA
A0A0A7KY21	Uncharacterized protein	9	47.2	21.226	4	ycfP
A0A0A7LAG3	DNA-binding transcriptional dual regulator of siderophore biosynthesis and transport	9	57.4	16.795	4	fur
A0A0A7KYZ0	Fused UDP-L-Ara4N formyltransferase/UDP-GlcA C-4-decarboxylase	8	10.9	74.288	11	arnA
A0A0A7L235	Iron-sulfur cluster assembly scaffold protein	8	53.1	13.848	3	iscU[8]
A0A0A7KWA5	Fused acetaldehyde-CoA dehydrogenase/iron-dependent alcohol dehydrogenase/pyruvate-formate lyase deactivase	7	8.6	96.126	9	adhE

A0A0A7L4R2	DNA-binding transcriptional repressor, Ni-binding	7	55.6	15.094	2	nikR
A0A0A7L3S2	Stationary phase protein, binds sigma 70 RNA polymerase subunit	7	46.8	18.243	1	rsd
A0A0A7L934	Putative CoA-binding protein	7	57.7	14.701	1	yccU
A0A0A7L068	CP4-6 prophage predicted dehydratase	6	12.2	69.398	10	yagF
A0A0A7L183	Putative transcriptional regulator, PadR family	6	29	23.401	8	yqjI
A0A0A7L1Y3	Dihydrodipicolinate synthase	6	21.2	31.27	5	dapA
A0A0A7L494	Isocitrate lyase	6	17.7	47.521	5	aceA[9]
A0A0A7KXX8	Pyruvate kinase II	6	16	51.357	4	pykA
A0A0A7L010	Cysteine desulfurase (TRNA sulfurtransferase), PLP-dependent	6	15.8	45.089	3	iscS
A0A0A7L755	Glutathione S-transferase homolog	6	31.7	22.545	3	yibF
A0A0A7L5S1	Alkyl hydroperoxide reductase, C22 subunit	6	42.2	20.761	2	ahpC
A0A0A7L278	30S ribosomal subunit protein S4	6	21.4	23.469	1	rpsD
A0A0A7L1Y9	30S ribosomal subunit protein S14	6	29.7	11.58	1	rpsN
A0A0A7L8G9	2-oxoglutarate decarboxylase, thiamin-requiring	5	5.6	105.06	10	sucA
A0A0A7KYQ9	D-tagatose 1,6-bisphosphate aldolase 2, subunit	5	11.7	47.108	8	gatZ
A0A0A7L058	Thioredoxin 2	5	40.3	15.555	6	trxC

A0A0A7L6X8	Thioredoxin reductase, FAD/NAD(P)-binding	5	15.9	34.623	4	trxB
A0A0A7L0S7	Fructose-bisphosphate aldolase, class II	5	20.9	39.147	4	fbaA[10]
A0A0A7KYJ8	D-tagatose 1,6-bisphosphate aldolase 2, catalytic subunit	5	21.5	30.812	4	gatY
A0A0A7L0P9	Lipid hydroperoxide peroxidase	5	44.6	17.835	3	tpx
A0A0A7L4E2	50S ribosomal subunit protein L14	5	35.8	13.541	2	rplN
A0A0A7L077	Glutamate-cysteine ligase	4	10	58.269	9	gshA
A0A0A7KWQ6	tRNA 2-thiocytidine biosynthesis protein	4	14.8	35.561	8	ttcA
A0A0A7L5K5	DNA-binding transcriptional dual regulator	4	11.3	33.384	5	araC
A0A0A7KZG1	NADH:ubiquinone oxidoreductase, chain E	4	25.3	18.59	5	nuoE[11]
A0A0A7L6B4	Pyridoxal phosphate (PLP) phosphatase	4	20.2	30.201	3	ybhA
A0A0A7L6E7	50S ribosomal subunit protein L2	4	18.7	29.86	2	rplB
A0A0A7L1S2	Putative enzyme IIB component of PTS	4	23.1	11.735	2	fryB
A0A0A7L1V5	30S ribosomal subunit protein S13	4	31.4	13.099	1	rpsM
A0A0A7L439	Thiamin phosphate synthase	4	31.2	15.656	1	yjbQ
A0A0A7L1W1	50S ribosomal subunit protein L5	4	21.8	20.301	1	rplE
A0A0A7L1V7	D-arabinose 5-phosphate isomerase	3	11.9	35.196	7	kdsD
A0A0A7L4U8	Phosphopentomutase	3	9.3	44.369	6	deoB

A0A0A7L417	6-phosphofructokinase I	3	11.2	34.842	6	pfkA[12]
A0A0A7L0M7	Colanic acid biosynthesis protein	3	7.5	47.343	5	wcaK
A0A0A7L707	Methionine sulfoxide reductase A	3	22.2	23.315	4	msrA
A0A0A7L625	Triosephosphate isomerase	3	7.8	26.972	3	tpiA
A0A0A7L222	Glyceraldehyde-3-phosphate dehydrogenase A	3	10.9	35.532	3	gapA[13]
A0A0A7L2T8	Glutaredoxin 3	3	27.7	9.1374	3	grxC[4]
A0A0A7L3P2	Sigma factor-binding protein, stimulates RNA polymerase holoenzyme formation	3	13.5	15.655	3	crl
A0A0A7KXQ8	Free methionine-(R)-sulfoxide reductase	3	19.4	18.121	3	msrC
B8LFD8	Galactoside O-acetyltransferase	3	5.4	22.799	2	lacA
A0A0A7L1X5	30S ribosomal subunit protein S11	3	22.5	13.845	2	rpsK[13]
A0A0A7L5S4	Peptidyl-prolyl cis-trans isomerase C (Rotamase C)	3	47.3	10.232	2	ppiC
A0A0A7L144	Aminomethyltransferase, tetrahydrofolate-dependent, subunit (T protein) of glycine cleavage complex	3	7.1	40.146	2	gcvT
A0A0A7L3R4	Primosome factor n (Replication factor Y)	2	3.1	81.654	11	priA
A0A0A7L866	Lipoate synthase	2	8.7	36.071	8	lipA
A0A0A7KYS5	DNA-binding transcriptional dual regulator, global regulator of anaerobic growth	2	9.2	27.967	5	fnr
A0A0A7L2E8	30S ribosomal subunit protein S12	2	12.1	13.71	4	rpsL[14]

A0A0A7L5W3	Pyrroline-5-carboxylate reductase, NAD(P)-binding	2	7.4	28.145	4	proC
A0A0A7KYV6	DNA-binding transcriptional regulator of rRNA transcription, DnaK suppressor protein	2	11.9	17.528	4	dksA
A0A0A7L338	Protein export chaperone	2	12.3	17.337	4	secB
A0A0A7L2V1	Galactitol-specific enzyme IIB component of PTS	2	28.7	10.222	4	gatB
A0A0A7L283	Fe/S biogenesis protein possible scaffold/chaperone for damaged Fe/S proteins	2	14.7	20.997	4	nfuA
A0A0A7L3K0	Putative cytoplasmic sugar-binding protein	2	7.2	15.292	3	rbsD
A0A0A7L1I8	Glutaredoxin-4	2	21.7	12.879	3	grxD
A0A0A7KX48	Iron-sulfur cluster insertion protein	2	18.4	12.1	3	erpA
A0A0A7L0C6	2-keto-3-deoxy gluconate (KDG) aldolase CP4-6 prophage	2	7.6	32.53	3	yagE
A0A0A7L2B9	30S ribosomal subunit protein S17	2	20.2	9.7043	2	rpsQ
A0A0A7L2X6	Putative acyltransferase with acyl-CoA N-acyltransferase domain	2	13	17.104	2	yiaC
A0A0A7L368	50S ribosomal subunit protein L28	2	12.8	9.0064	1	rpmB
A0A0A7L6A1	50S ribosomal subunit protein L17	2	8.7	14.364	1	rplQ
A0A0A7L1H3	30S ribosomal subunit protein S21	2	14.1	8.4999	1	rpsU

MsrA, MsrC, Tpx, and AhpC are well-known substrate proteins of *E. coli* Trx1. Genes for proteins that are known to interact with Trx in other organisms are labeled with reference numbers, and the references are listed in Supplementary Reference below.

Supplementary Reference

1. Nair, R.N., Lee, P.J., Rheingold, A.L. & Grotjahn, D.B. Single bifunctional ruthenium catalyst for one-pot cyclization and hydration giving functionalized indoles and benzofurans. *Chem. Eur. J.* **16**, 7992-7995 (2010).
2. Walker, W.H. & Rokita, S.E. Use of a boroxazolidone complex of 3-iodo-l-tyrosine for palladium-catalyzed cross-coupling. *J. Org. Chem,* **68**, 1563-1566 (2003).
3. Marchand C., Le Marechal P., Meyer Y., Decottignies P. Comparative proteomic approaches for the isolation of proteins interacting with thioredoxin. *Proteomics* **24**, 6528–37 (2006).
4. Alkhalfioui F., Renard M., Vensel W.H., Wong J.H., Tanaka C.K., Hurkman W.J., et al. Thioredoxin-linked proteins are reduced during germination of seeds of Medicago truncatula. *Plant Physiol.* **144**, 1559–79 (2007).
5. Balmer Y., Vensel W.H., Cai N., Manieri W., Schürmann P., Hurkman W.J., et al. A complete ferredoxin/thioredoxin system regulates fundamental processes in amyloplasts. *Proc. Nat. Acad. Sci. USA* **103**, 2988–93 (2006).
6. Yamazaki D., Motohashi K., Kasama T., Hara Y., Hisabori T. Target proteins of the cytosolic thioredoxins in Arabidopsis thaliana. *Plant Cell Physiol.* **45**, 17–24 (2004).
7. Pérez-Pérez M.E., Florencio F., Lindahl M. Selecting thioredoxins for disulphide proteomics: target proteomes of three thioredoxins from the cyanobacterium Synechocystis sp. *Proteomics* **6**, S186–S195 (2006).
8. Ding H., Harrison K., Lu J. Thioredoxin reductase system mediates iron binding in IscA and iron delivery for the iron-sulfur cluster assembly in IscU. *J. Biol. Chem.* **280**, 30432-7 (2005).
9. Lemaire S.D., Guillon B., Le Marechal P., Keryer E., MiginiacMaslow M., Decottignies P. New thioredoxin targets in the unicellular photosynthetic eukaryote Chlamydomonas reinhardtii. *Proc. Nat. Acad. Sci. USA* **101**, 7475–80 (2004).
10. Wong J.H., Balmer Y., Cai N., Tanaka C.K., Vensel W.H., Hurkman W.J., et al. Unraveling thioredoxin-linked metabolic processes of cereal starchy endosperm using proteomics. *FEBS Lett.* **547**, 151–6 (2003).
11. Balmer Y., Vensel W.H., Tanaka C.K., Hurkman W.J., Gelhaye E., Rouhier N., et al. Thioredoxin links redox to the regulation of fundamental processes of plant mitochondria. *Proc. Nat. Acad. Sci. USA* **101**, 2642–7 (2004).
12. Funato Y., Hayashi T., Irino Y., Takenawa T., Miki H. Nucleoredoxin regulates glucose metabolism via phosphofructokinase. *Biochem. Biophys. Res. Commun.* **440**, 737-42 (2013).
13. Lemaire S.D., Guillon B., Le Marechal P., Keryer E., MiginiacMaslow M., Decottignies P. New thioredoxin targets in the unicellular photosynthetic eukaryote Chlamydomonas reinhardtii. *Proc. Nat. Acad. Sci. USA* **101**, 7475–80 (2004).
14. Wong J.H., Cai N., Balmer Y., Tanaka C.K., Vensel W.H., Hurkman W.J., et al. Thioredoxin targets of developing wheat seeds identified by complementary proteomic approaches. *Phytochemistry* **65**, 1629–40 (2004).
